# Transcriptome of Extracellular Vesicles Released by Hepatocytes

**DOI:** 10.1371/journal.pone.0068693

**Published:** 2013-07-11

**Authors:** Felix Royo, Karin Schlangen, Laura Palomo, Esperanza Gonzalez, Javier Conde-Vancells, Agustin Berisa, Ana M. Aransay, Juan M. Falcon-Perez

**Affiliations:** 1 Metabolomics Unit, CIC bioGUNE, CIBERehd, Derio, Spain; 2 Genome Analysis Platform, CIC bioGUNE, CIBERehd, Derio, Spain; 3 R&D and Innovation Department, FAES FARMA S.A., Leioa, Spain; 4 IKERBASQUE, Basque Foundation for Science, Bilbao, Spain; Wageningen UR Livestock Research, The Netherlands

## Abstract

The discovery that the cells communicate through emission of vesicles has opened new opportunities for better understanding of physiological and pathological mechanisms. This discovery also provides a novel source for non-invasive disease biomarker research. Our group has previously reported that hepatocytes release extracellular vesicles with protein content reflecting the cell-type of origin. Here, we show that the extracellular vesicles released by hepatocytes also carry RNA. We report the messenger RNA composition of extracellular vesicles released in two non-tumoral hepatic models: primary culture of rat hepatocytes and a progenitor cell line obtained from a mouse foetal liver. We describe different subpopulations of extracellular vesicles with different densities and protein and RNA content. We also show that the RNA cargo of extracellular vesicles released by primary hepatocytes can be transferred to rat liver stellate-like cells and promote their activation. Finally, we provide *in vitro* and *in vivo* evidence that liver-damaging drugs galactosamine, acetaminophen, and diclofenac modify the RNA content of these vesicles. To summarize, we show that the extracellular vesicles secreted by hepatocytes contain various RNAs. These vesicles, likely to be involved in the activation of stellate cells, might become a new source for non-invasive identification of the liver toxicity markers.

## Introduction

In recent years, intercellular transference of active macromolecules mediated by cell-released extracellular vesicles (EVs) has become a recognized key regulatory mechanism in a growing number of biological processes including development, cancer, immunity and inflammation [1], [2], [3]. There are various mechanisms of formation of these vesicles, creating a complex repertory of EVs [4]. The vesicles can be formed by outward budding from the plasma membrane giving origin to so-called microparticles, microvesicles, shedding particles or ectosomes [5]. Inward budding of the membrane of endocytic organelles generates the multivesicular bodies which contain another kind of vesicles, called exosomes. Exosomes are released to extracellular media by the fusion of multivesicular bodies to the plasma membrane [4]. Independently of their biogenesis, EVs carry lipids, proteins, and nucleic acids: both coding and non-coding RNAs [6], [7]. A wide range of cells, of either tumoral or non-tumoral origin, can release EVs to the culture media [8]. These EVs can be then captured by other cells that will accept their cargo and, as a consequence, will undergo modifications according to the encoded signals [9], [10], [11], [12], [13]. EVs have been also detected in biological fluids such as blood, urine, and ascitic fluid [14]. Thus, they have the potential to release their cargo in both paracrine and long distance manner; this feature is being widely exploited in non-invasive disease biomarker discovery.

The extensive proteomic analysis of EVs in different cellular systems have found some common as well as cell-type-specific proteins [14]. In contrast, vesicle RNA content has not been examined so widely, and in particular, very little is known about vesicle messenger RNAs (mRNAs). Initial studies of mast [13] and glioma [12] cells have shown the presence of both messenger and microRNAs in the EVs. The subsequent research concentrated mostly on the microRNAs, probably because of their direct involvement in epigenetic regulatory processes [15], [16].

Our group has previously reported that hepatic non-tumoral cells and primary culture of hepatocytes can release EVs, including exosomes [17]. We also performed the proteome analysis of EVs, finding a large number of enzymes involved in endogenous and xenobiotic metabolism [17], [18]. These findings suggest that the vesicles might be involved in response to stress conditions in the liver. In the current work, we characterized the messenger RNAs present in the EVs released in two hepatic cellular models using microarray technology. We found that RNA content depends on the cellular model used. We compared these different RNA sets to other, already catalogued, messenger RNAs from various non-tumoral cell types. As a result, we obtained a set of 223 RNAs associated mainly with hepatocyte functions. Remarkably, we found that some of those RNAs are enriched in the EVs in comparison with the intracellular transcriptome. This result suggests the existence of a regulated sorting mechanism to control loading of specific transcripts into the EVs. We also validated some of the RNAs by quantitative real-time PCR (qPCR) and demonstrated that the RNA component is incorporated into and affects the recipient cells. Finally, using *in vitro* and *in vivo* liver disease models, we demonstrated that the hepatic RNA-containing EVs are a suitable biological source for non-invasive biomarker discovery.

## Materials and Methods

### Reagents, Cell Culture

All media and reagents for tissue culture were purchased from GIBCO (Life Technologies Inc.). MLP29 is a murine liver progenitor cell line [19]. The cell line called 8B is a rat myofibroblastic hepatic stellate cell line (HSC) [20]. Analytical grade reagents were mostly acquired from Sigma-Aldrich (St. Louis, MO). Mouse monoclonal antibodies were purchased from several vendors: anti-AIP1, anti-flotillin (clone 18) from BD Biosciences (Mountain View, CA, USA), and anti-Tsg101 (clone 4A10) from Abcam (Cambridge, UK). Armenian hamster anti-mouse Cd81 (clone Eat2) was purchased from Serotec (Oxford, UK).

### Animal Procedures

All animal experimentation was conducted in accordance with Spanish guidelines for the care and use of laboratory animals, and protocols approved by the CIC bioGUNE ethical review committee (Permit Number: P-CBG-CBBA-3610). All surgery was performed under anaesthetic gas Isoflurane (IsoFLO, Abbott Laboratories), and all efforts were made to minimize suffering. Rat hepatocytes (RH) were obtained by liver perfusion of nine-week-old healthy Sprague-Dawley male rats. For *in vivo* model of liver damage, rats received an intraperitoneal injection of 1 g/kg/5 ml of D(+)-galactosamine hydrochloride (GalN, Sigma-Aldrich). A control group of animals received the same volume of saline solution. Blood samples from each animal were drawn 18 h after the injection, and the sera were transferred to fresh Eppendorf tubes and stored at −80°C.

### Production and Purification of EVs

To obtain EVs, MLP29 cells or fresh RH suspension were plated in non-collagenized (MLP29) or collagen-coated (RH) 150 -mm dishes, at 15–30 million cells per dish. Cells were cultured in complete DMEM medium [Dulbecco’s modified Eagle medium supplemented with 10% (v/v) foetal bovine serum (FBS), 0.1 mg/ml streptomycin, and 100 units/ml penicillin (GIBCO, Life Technologies Inc.)] for 24 h (MLP29 cells) or for 4 h (RH), at 37°C and 5% of CO_2_. The cells were washed twice with Dulbecco’s modified phosphate-buffered saline (PBS) and incubated for 48 h (MLP29 cells) or 36 h (RH) in 25 mM HEPES-containing complete DMEM medium (contaminating vesicles were first removed by overnight centrifugation at 110000×g [21]). In cases of hepatotoxic drug treatment, the drugs were added to the vesicle-depleted media. The concentrations employed were: 10 mM galactosamine (Sigma-Aldrich), 10 mM acetaminophen (Sigma-Aldrich), or 400 µM diclofenac (Sigma-Aldrich). After incubation, media were collected, and EVs were isolated as previously described [22]. Briefly, culture supernatant was centrifuged at 1500×g for 10 min to remove lifted cells and cellular debris. The resultant supernatant was filtered through 0.22 µm-pore filters, followed by ultracentrifugation at 10000×g and 100000×g for 30 min and 60 min, respectively. The resulting pellets were resuspended in PBS, pooled, and again ultracentrifuged at 100,000×g for 60 min. The final pellet of EVs was resuspended in PBS to between 1/700-th and 1/2000-th of the original volume of the culture supernatant, and the aliquoted solution was stored at −80°C. To purify the EVs further, sucrose density gradients ranging from 2.0 to 0.25 M sucrose in HEPES were prepared [21]. When indicated, the purification was performed using Exoquick (SBI System Biosciences) in accordance with the manufacturer’s protocol. Briefly, samples of serum or cell media were pre-cleared by centrifugation at 3000 g for 30 min and then mixed with 1 ml of Exoquick per 1 ml of media or 63 µl per 250 µl of serum. After that, the samples were incubated at 4°C overnight and centrifuged at 1500×g for 30 min.

### EVs Capture and Hepatic Stellate Cell Line Activation Assays

Capture experiments were conducted using the myofibroblastic rat HSC 8B [20]. The cells were incubated in 6-well plates at 50 000 cells per well for 12 h, and then the medium was replaced with OptiMEM (GIBCO, Life Technologies Inc.) supplemented with 2% FBS. To standardize assays, total protein concentration from EV preparations was measured by Bradford assay (Bio-Rad Laboratories Inc). An aliquot (5 µg) of EVs was diluted in 1 ml OptiMEM-2% FBS and filtered through filters with 0.22 µm pore diameter before incubation with the cells for the indicated time periods. As control, EVs were treated with RNase as described below. At the end of incubation periods, cells were washed three times with PBS, then scraped in PBS and centrifuged at 1000×g for 10 min. Cell pellets were kept frozen at −80°C until RNA extraction and qPCRs were performed as described below.

To follow the capture of EVs through immunofluorescence, 80 ug of RH-derived EVs were labelled with DiI Vibrant-cell labelling solution (Life Technologies, Inc). The label reaction took place in 1 ml of PBS with 5 ul of cell labelling solution for 1 h at 37°C. After the reaction, EVs were recovered using a sucrose-cushion as described in [23]. When indicated, EVs were treated with Tx-100 and RNase as described below. Labelled EVs were given to 8B cells cultured on glass covers, in the same amount and condition than described above. As a negative control, the same amount of label solution without EVs was subjected to sucrose-cushion purification, and added to cells.

### RNAse Protection Assays

To test the ability of EVs to protect against RNase activity, crude free human RNA (hRNA) was mixed with EVs preparations and RNase A (100 ng/µl), and incubated for 15 min at 37°C, with or without 0.1% Tx-100.

### Electron Microscopy

For cryo-electron microscopy, EV preparations were directly adsorbed onto glow-discharged holey carbon grids (QUANTIFOIL, Germany). Grids were blotted at 95% humidity and rapidly plunged into liquid ethane with the aid of VITROBOT (Maastricht Instruments BV, The Netherlands). Vitrified samples were imaged at liquid nitrogen temperature using a JEM-2200FS/CR transmission cryo-electron microscope (JEOL, Japan) equipped with a field emission gun and operated at an acceleration voltage of 200 kV.

### Nanoparticle Tracking Analysis (NTA)

Size distribution within EV preparations was analyzed by measuring the rate of Brownian motion using a NanoSight LM10 system (NanoSight, Amesbury, U.K.), which is equipped with a fast video capture and particle-tracking software. NTA post-acquisition settings were kept constant for all samples, and each video was analyzed to give the mean, mode, and median vesicle size, and an estimate of the concentration [24].

### RNA Isolation, Characterization, and Gene Expression Arrays

Total RNA isolation was achieved by two different methods. Total RNAs for microarray hybridization were extracted using TRIzol reagent (Life Technologies, Inc), and their integrity, size and quantification were evaluated in RNA 6000 Pico Chips with a Bioanalyzer (Agilent Technologies). Subsequently, cRNAs were prepared using 0.3–0.5 ug of total RNA obtained from 250 millions of cells, according to manufacturers’ recommendations and hybridized on MouseWG-6 v2.0 Expression BeadChips (Illumina, Inc) in case of the murine MLP29 RNA, and on Genechips Rat Genome 230 2.0 [Rat230_2] (Affymetrix®) for the rat RH RNA. For array validation, characterization, gradients, and cell capture, RNA was isolated using RNeasy columns (Quiagen, Inc).

### Reverse Transcriptase, PCR, and qPCR

cDNA was synthesized from 0.1–0.5 µg of RNA using the Quanta cDNA Supermix (Quanta Inc) following the manufacturer’s recommendations. To amplify gene fragments, PCR was performed according to the manufacturer’s protocol for *Taq* polymerase (Biotools). For relative quantification of genes, qPCR was performed in duplicates using SYBR Green PCR Master Mix ((Bio-Rad Laboratories Inc) ) in an iCycler thermocycler (Bio-Rad Laboratories Inc). Primers were designed using Primer3 [25], and the sequences are listed in Table S1. The efficiency of each set of primers was determined by serial dilution of the template cDNA. The theoretical value obtained with the iCycler software was 100±5% (Efficiency (%) = 100×[10(−1/slope) –1]; Bio-Rad Laboratories Inc).

To confirm the array results, three independent samples were analyzed by qPCR for MLP29 and fold-change in gene expression calculated using *Rplp0* as reference gene, according to the formula: 2 - ΔΔCt. In the experiments examining the stellate cell activation, we calculated the increase in nitric oxide synthase 2 (*Nos2)* transcript levels after EVs treatment using 28S rRNA gene as a reference. Since no amplification for *Nos2* was observed in the untreated cells, mRNA level in EVs +0.1% Tx-100 samples was taken as a control value.

### Western Blot Analysis

The protein concentration in the preparations was determined by Bradford protein assay, using Bovine Serum Albumin (BSA) as standard. Samples were incubated for 5 min at 37°C, 65°C and 95°C and separated on 4–12% pre-casted gels from Life Technologies, Inc. After the transfer to nitrocellulose membranes, the samples were blocked overnight (5% milk and 0.05% Tween-20 in PBS), and the primary antibody was added for 1 h, followed by PBS wash and the application of secondary HRP-conjugated antibody. All proteins were detected under non-reducing conditions. Chemiluminescence detection of bands was performed using ECL Plus reagent (GE Healthcare, Buckinghamshire, UK).

### Gene Expression Array Data Analyses

Raw expression data were log_2_-transformed and quartile-normalized using the *lumi* tool [26] in R/Bioconductor statistical computing environment (http://www.bioconductor.org/). The data for the probes with a detection *p*-value higher or equal to 0.01 were excluded. The data detected with *p*-value lower than 0.01 in at least one array were accepted as significant. Thus, the data for the probes that did not pass the test were filtered out, and the genes they represented were considered to be “not expressed”.

For the detection of differentially expressed genes, a linear model was fitted to the data and empirical Bayes moderated t-statistics were calculated using the *limma* package [27] from Bioconductor. P-values were adjusted using Benjamini and Hochberg’s False Discovery Rate (FDR) Method.

To assess biological processes affected, the identified transcripts were submitted to Ingenuity Pathway Analysis (IPA) program to perform functional analysis or canonical pathway analysis. Functional analyses identify functions that are significantly enriched in the data set. Right-tailed Fisher’s Exact Test was used to calculate *p*-values for determining the probability of each biological function assigned to the data set to be due to chance alone. Ingenuity-defined canonical pathways that were most significantly enriched in the dataset were also reported. In this case, Fisher’s Exact Test was used to calculate the probability that the association between the genes in the dataset and the canonical pathways can be explained by chance alone.

### Statistical Analysis

To calculate statistical correlation between qPCR and array data, r^2^ coefficient of correlation and the associated *p*-value were calculated using Prism5 (GraphPad Software Inc.). Using the same software, ANOVA analysis with Bonferroni post-hoc test was performed to calculate the differences between *Nos2* expression levels after different cell treatments. One asterisk denotes statistical significance (p<0.05).

## Results

### EVs Released by Non-tumoral Hepatic Cells Carry their Own Transcriptome

**Figure 1:**
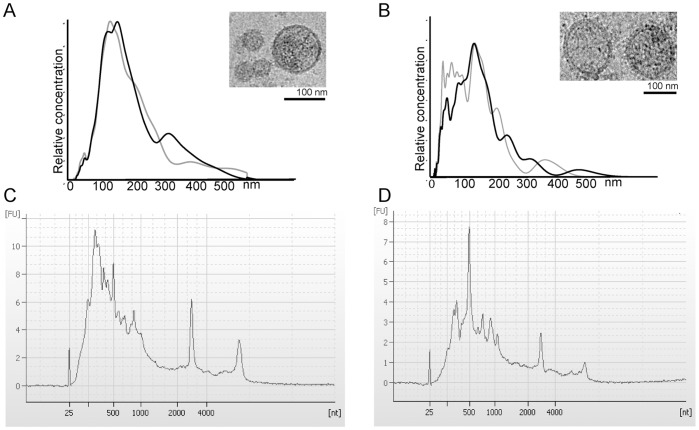
Characterization of EVs. Characterization of EVs from MLP29 (A, C) and rat primary hepatocytes (RH) (B, D). The NTA analyses of two independent samples for each cell type show more heterogeneous vesicle populations released by primary culture of hepatocytes. Cryo-TEM pictures show membrane vesicles of different sizes (insets A–B). Bioanalyzer profiles of total RNA extracted by RNeasy from both cell types are similar with a wide distribution on size and also the presence of a reduced amount of ribosomal RNAs (C–D).

First, we looked for RNA in EVs purified from two non-tumoral hepatic cellular models – a murine cell line MLP29 and primary culture of rat hepatocytes – as described previously [17]. Cryo-electron microscopy and nanoparticle tracking (NTA) analyses revealed that both cell types contained heterogeneous EV populations with diameters between 50 and 500 nm although the majority were smaller than 250 nm (**Figure 1A–B**). RNA from these EVs preparations was extracted using TRIzol reagent, which performs well with this type of samples [28]. Bioanalyzer analysis of the extracts (**Figure 1C–D**) clearly showed that EVs released by non-tumoral hepatocytes contain RNA transcripts of different lengths and a reduced amount of the ribosomal fraction, in agreement with the data obtained from other cell types [12], [13], [29], [30], [31], [32]. The amount of RNA relative to protein content associated with EVs was higher in the murine MLP29 cellular model than in the primary culture of rat hepatocytes, with average values of 3 and 0.5 ng of RNA per µg of protein, respectively. Roughly, a million MLP29 cells produced 0.9 ng (SD +/−0.67, n = 3) of RNA and the same number of primary RH cells yielded 1.2 ng (SD +/−0.60, n = 3).

**Figure 2:**
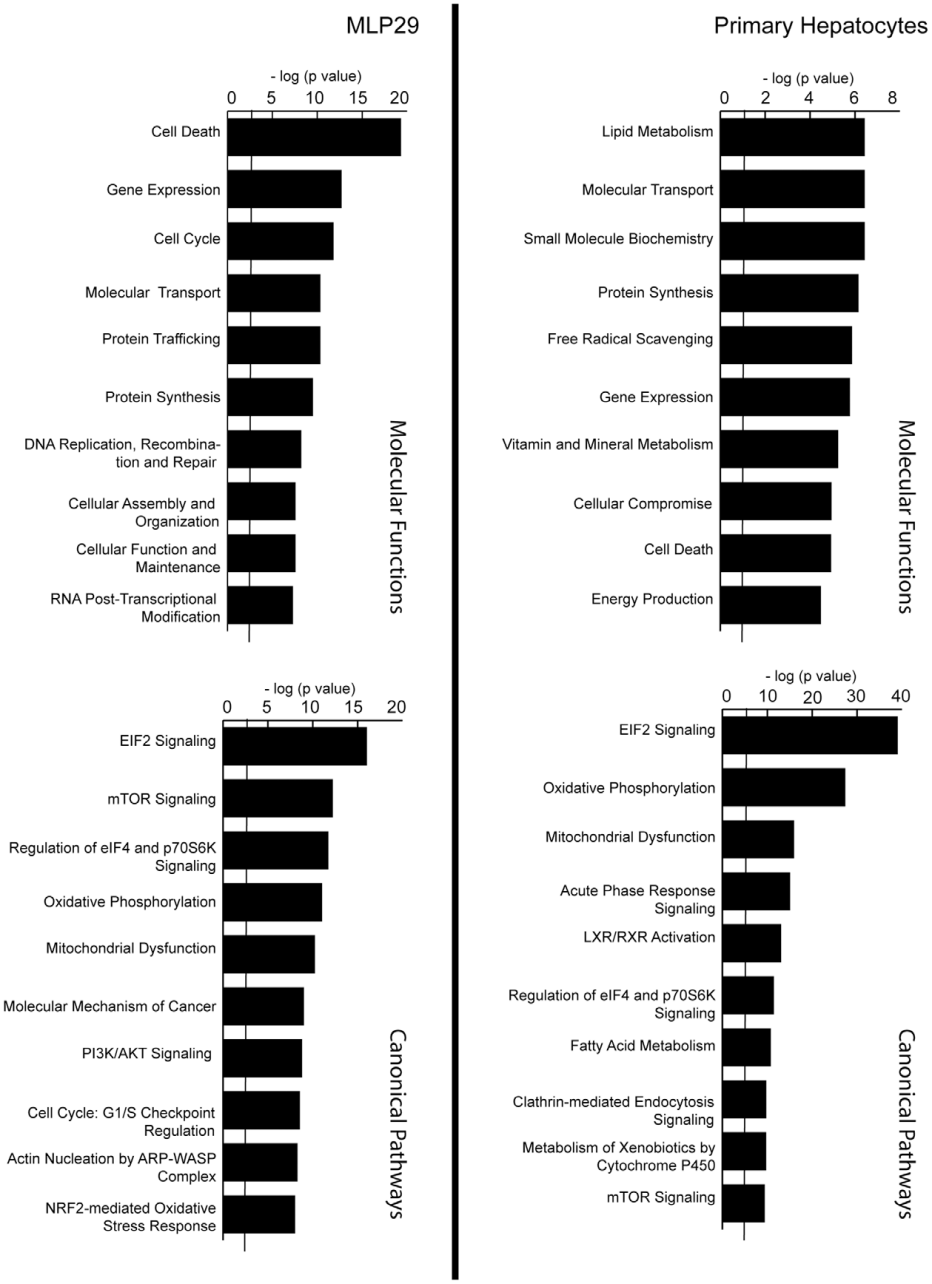
Comprehensive Pathway analysis of EVs transcriptome. Ingenuity Pathway Analysis of transcripts detected in EVs released by non-tumoral hepatic cellular models.

Next, we characterized the transcripts found in these vesicles by performing a microarray expression analyses as described in Materials and Methods, using the Illumina MouseWG-6 v2.0 Expression BeadChip for the murine MLP29 cells and Affymetrix GeneChip Rat Genome 230 2.0 [Rat230_2] for the rat model (primary hepatocyte culture). These microarray analyses were deposited at the MIAME-express repository with ID number E-MEXP-3740. A total of 6512 and 1336 transcripts were above the significance threshold in the murine and rat models, respectively; the sets shared 682 transcripts. The analysis of the molecular functions represented by the transcriptome of each cell model reflects the expected discrepancy due to their different cellular origins. While EVs from adult primary hepatocytes are enriched in transcripts related to metabolism (lipids, small molecules, vitamins) and energy production, EVs from the embryonic liver-derived cell line are enriched in transcripts that encode proteins associated with cell death, cell cycle, and cellular organization (**Figure 2**). The analysis of the canonical pathways provided by IPA program also confirms enrichment in transcripts linked to metabolic pathways in primary hepatocyte-derived EVs in comparison with EVs from MLP29 cell line. The MLP29 EVs are enriched in transcripts from proliferation-related pathways, including mTOR and PI3K/AKT signalling (**Figure 2**).

**Figure 3:**
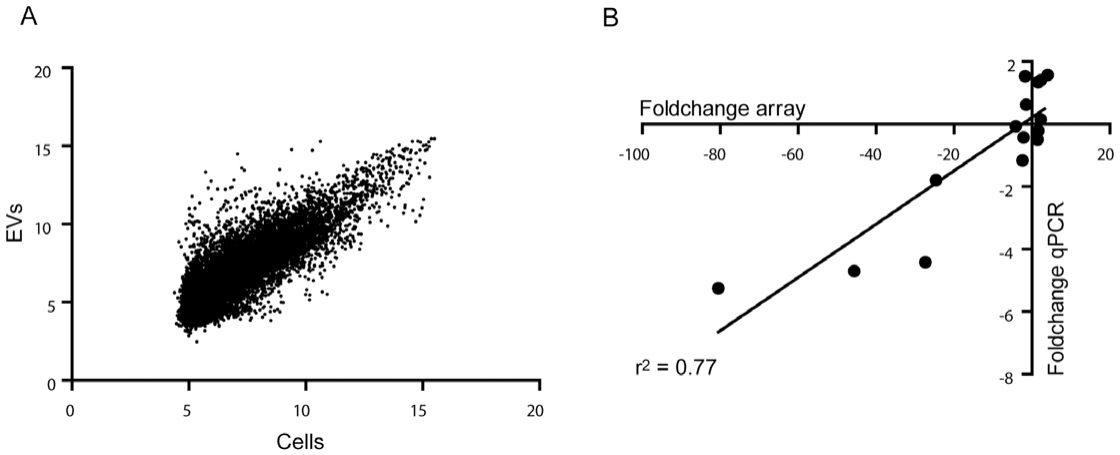
Comparison between cellular and EVs transcriptomes in MLP29 cellular model. (A) Plot of fluorescent intensities for MLP29 EVs and cells reveals a group of genes enriched or underrepresented in EVs. (B) Plot shows the correlation of the fold changes estimated by microarray analysis and the fold change calculated by qPCR for a set of genes.

The gene expression at the whole genome level (microarrays) was only characterized for one sample per condition. To confirm the observed expression level trends in a set of transcripts, we performed qPCR for RNAs from three independent EV preparations of each model. The transcripts were chosen by their distinct intensity signal in the RH data (primary hepatocyte, **Tables SI and S2**) or by their differential enrichment in EVs compared to intracellular RNA content (MLP29 cell line, **Figure 3A**, **Table S3**). The Ct values in the qPCR analysis for each cellular model correlated (r^2^ = 0.52, *p* = 0.005 and r^2^ = 0.41, *p* = 0.02 for primary hepatocytes and MLP29 cells, respectively) with the tendencies of the intensity signal measured in the microarray expression analysis (Figure S1). For the MLP29 cell line, the microarray analysis also included the comparison with the intracellular transcripts present in those cells. Thus, 8856 transcripts were detected above the significance threshold in these cells, and among them, 6345 were detected also within MLP29-released EVs. Out of these common transcripts, 1673 were under-represented, while 1930 were significantly enriched in EVs (fold-change value higher than 2, *p*<0.001). To confirm the fold-changes for some genes (see **Table SI**), qPCR for cells and EVs were carried out. The results showed a high correlation (r^2^ = 0.77, *p* = 0.0001) with the drift observed in the microarray expression analysis (**Figure 3B**).

To perform subsequent experiments, a subset of genes was chosen according to their enrichment or abundance in EVs. In the case of MLP29, the subset includes acidic (leucine-rich) nuclear phosphoprotein 32 family, member B (Anp32b), neuroepithelial cell transforming 1 (Net1), and trafficking protein, kinesin binding 2 (Trak2). In the case of EVs derived from RH, we followed albumin (Alb) and retinol binding protein 4, plasma (Rbp4). Moreover, due to the relevance in the biological context of the liver function, we also followed cytochrome P450, family 2, subfamily d, polypeptide 1 (Cyp2d1),cytochrome P450, family 2, subfamily e, polypeptide 1 (Cyp2e1), and guanine nucleotide binding protein (G protein), beta polypeptide 2-like 1 (Gnb2l).

**Figure 4:**
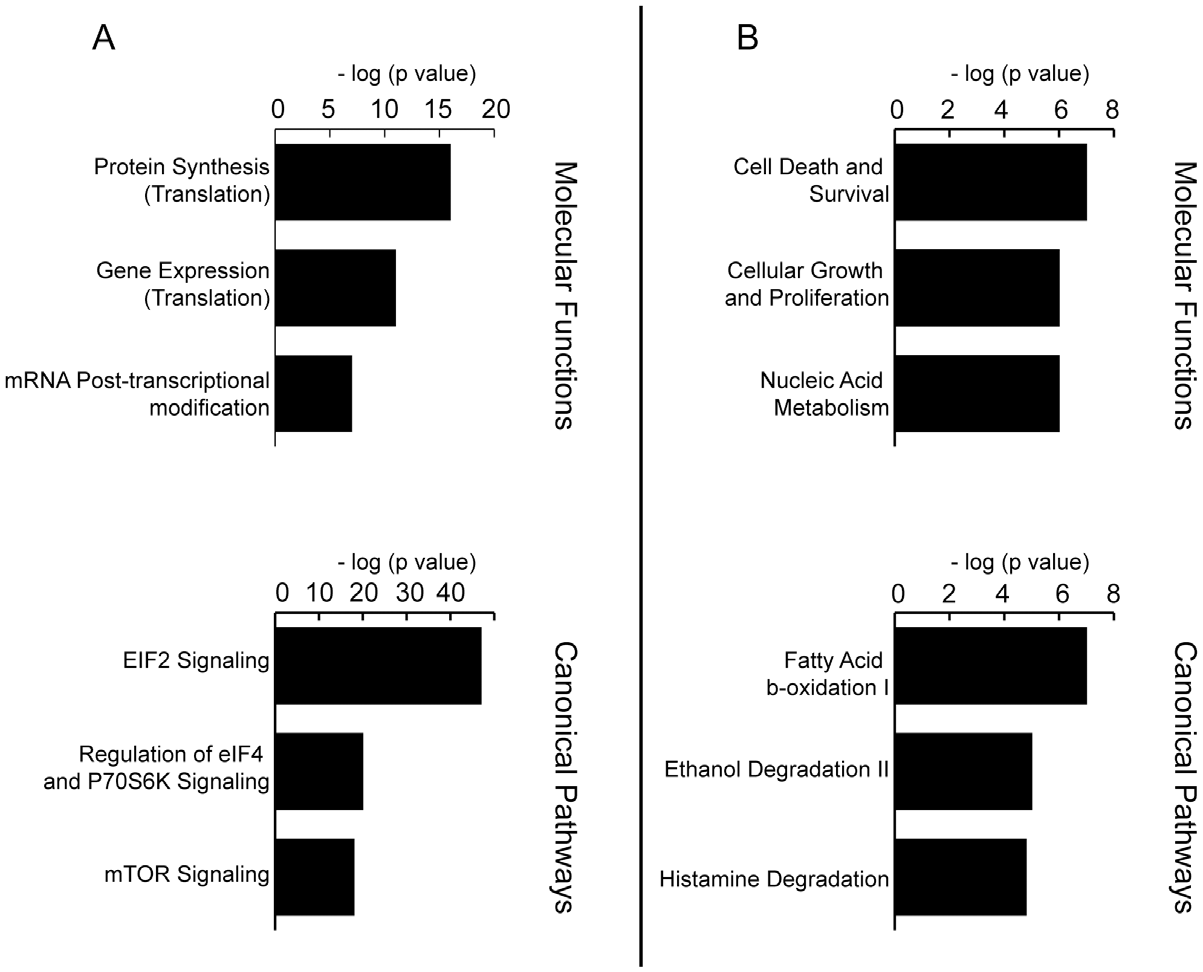
Comparison between the transcriptomes from MLP29 and RH’s EVs, and other published transcriptomes. Published transcriptomes from endothelial, mesenchymal [13] and cardiomyocyte [33] EVs. The common genes among the five cell types (A) are enriched in translation and ribosomal machinery categories. For the transcripts only detected MLP29 and RH (B), we found enrichment in pathways related to fatty acid metabolism and ethanol degradation.

When our results are compared with other transcriptomes reported for EVs of non-tumoral cellular models such as mast cells [13], cardiomyocytes [33], and endothelial cells [34], 43 transcripts appear to be common to all those lists (**Table S4**), and 223 are unique to our analysis (**Table S5**). IPA analysis of common transcripts for all EV types (**Figure 4A**) showed a shared background in protein synthesis and transcription machinery. In addition, this analysis showed that the transcripts detected only in the EVs from cell lines of hepatic origin are involved in fatty acid metabolism and ethanol degradation (**Figure 4B**).

### EV Protection Against RNase Activity and the Presence of Full Coding Regions of Transcripts

**Figure 5:**
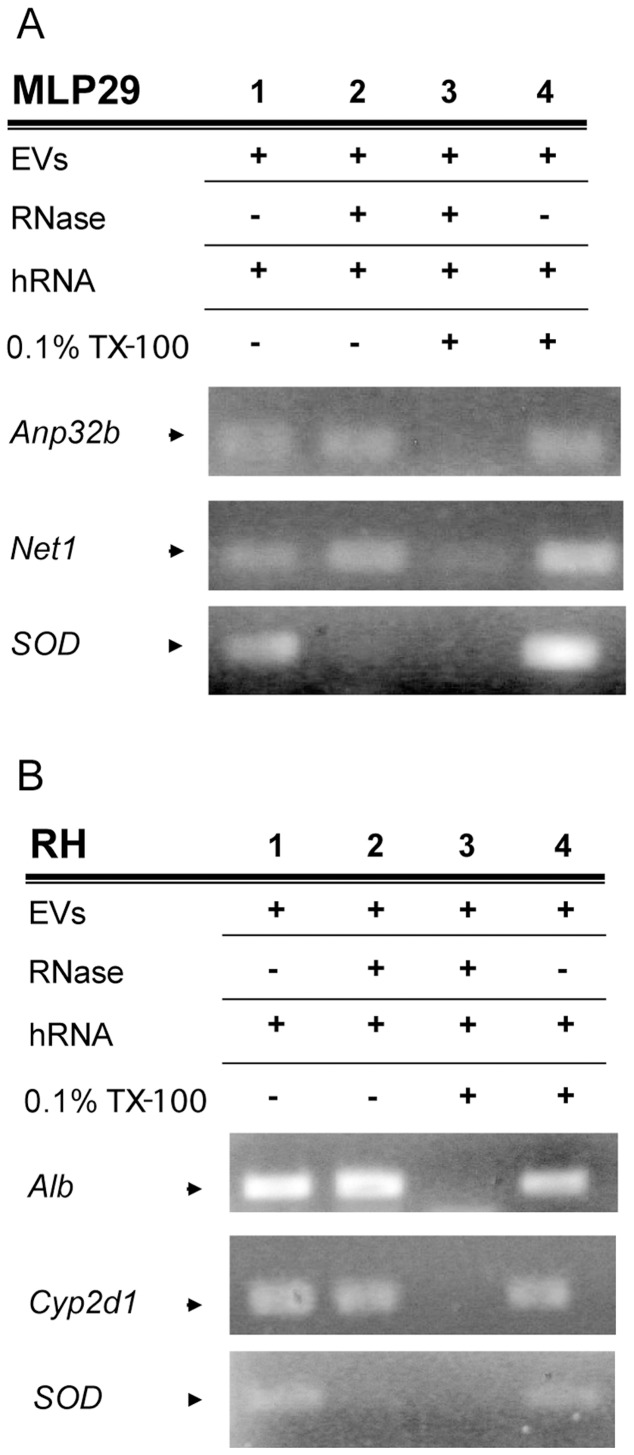
RNase protection assay. Both MLP29 (A) and RH (B) transcripts can be detected even if RNase activity is present. We added 1 µg of human RNA to 10 µg of EVs in PBS solution, and examined the extracted RNA samples for the presence of vesicular or human RNA (transcript *SOD* coding for human superoxide dismutase protein) (lane 1). In the suspensions treated with RNase (lanes 2 and 3), the added human RNA was degraded. The transcripts in EVs were only degraded if detergent had been added to the solution to disrupt the vesicular membrane (lane 3). The detergent did not interfere with RNA extraction and subsequent PCR (lane 4).

The RNase protection feature reported for EVs [13] was also confirmed for MLP29- and primary RH-derived EVs. This was demonstrated by the PCR amplification in RNase-treated EVs obtained for *Anp32b* and *Net1* (enriched in MLP29-released EVs) or *Alb* and *Cyp2d1* (highly represented in primary RH-released EVs) transcripts (**lanes 2** **Figure 5A–B**). The presence of active RNase in the sample was confirmed by the addition of exogenous total human mRNA; the amplification of the gene encoding hSOD protein was achieved only in the RNase-untreated samples (**lanes 1, 4,** **Figure 5A–B** **)**. The permeabilization of EVs using detergent showed that the EV RNAs are susceptible to RNase activity **(lanes 3,** **Figure 5** **A–B)**. To judge the integrity of transcripts from EVs, we examined the coding region of *Anp32b* gene in both cell models (Figure S2). We were able to amplify a fragment containing the full coding sequence for the protein, confirming the presence of full-length transcripts in these EVs.

### Primary Hepatocytes Release a Heterogeneous Population of EVs

**Figure 6:**
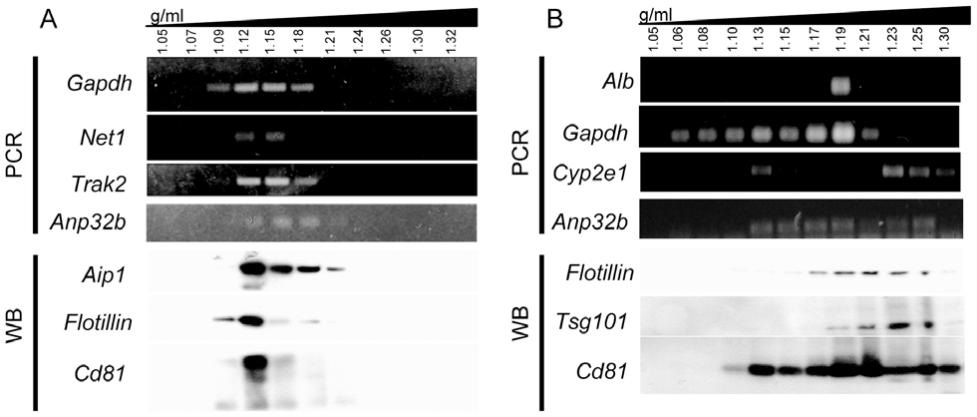
Density fractionation of EVs. The figure shows sucrose gradients of EVs preparations from MLP29 (A) and RH (B). Aliquots of these fractions were used for RNA extraction and protein extraction; the most abundant transcripts were found in the fractions containing typical exosomal markers (Tsg101 or Aip1). RH preparations showed more diversity, with vesicle populations fractionating at different densities.

To examine the vesicle subpopulations in EVs preparations, sucrose gradients were used as previously described [35]. The protein markers associated with exosomes lay mainly between 1.09 and 1.21 g/ml fractions, matching the most abundant transcripts identified in MLP29-released EVs (**Figure 6** **A**). In contrast, the profiles of primary RH-derived EVs were heterogeneous, in agreement with NTA analysis (**Figure 1B**), and different subpopulations could be defined (**Figure 6B**). A *Cd81*-containing fraction with density of 1.13 g/ml carried detectable amounts of *Cyp2e1*, *Gapd,* and *Anp32b*, but not *Alb* transcripts. Another population of vesicles enriched in *Alb* and *Gadph* transcripts was detected in a fraction with density of 1.19 g/ml. This fraction also contained high levels of Cd81 and Flotillin, and low, although detectable, levels of the exosomal protein marker Tsg101. The third EV population, with density of 1.23 g/ml, was enriched in Tsg101 protein and contained detectable levels of Cd81 and Flotillin proteins. This population also contained *Cyp2e1*, but not *Alb* or *Gadph* transcripts. Other populations containing *Gadph* were also observed (**Figure 6B**). Overall, these results showed differences between EVs released by different cell types and revealed the complex composition of EVs released by primary hepatocytes.

### Activation of Hepatic Stellate Cells Mediated by RNA-containing EVs from Primary Hepatocytes

**Figure 7:**
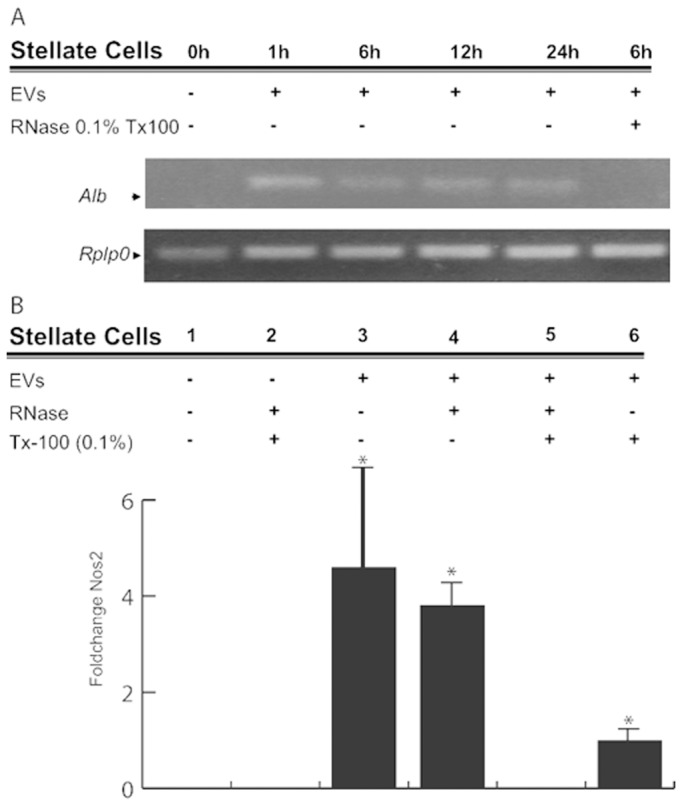
Hepatic stellate cells capture hepatocyte-released EVs and become activated. HSC 8B cells capture EVs from RH and respond to these stimuli by increasing *Nos2* transcription. (A) The capture can be followed by detecting the presence of *Alb*, an RH-specific transcript, for up to 24 hours after capture. When EVs were treated with RNase in the presence of detergent before incubation with the cells, *Alb* transcript was not detected (6 h incubation time), indicating that the transcript must have been transferred from the hepatocyte-released EVs. (B) The activation of HSCs can be followed by the expression of the protein nitric oxide synthase 2 (Nos2). *Nos2* is expressed at a very low level in HSC 8B (lanes 1 and 2), and clearly expressed if the cells are treated with EVs (lane 3 and 4), as previously described (31). After incubation with EVs pre-treated with RNase in the presence of detergent, we do not detect *Nos2* transcription (lane 5). HSC 8B cells incubated with EVs pre-treated only with detergent increase the transcription of *Nos*2 (lane 6), although to lower levels than the cells treated with intact EVs (lanes 3 and 4). In the graph, error bars represent SD (n = 2),* denotes p<0.05 respect to control, lane 1.

Liver contains various cell types that need to be finely coordinated in order to respond to different stimuli and stress conditions that continuously assault this essential organ [36]. HSCs belong to a liver-specific cell type with a major role in fibrosis, an integrated response to liver injury and stressful conditions [37]. To study the potential role of RNA carried in EVs released by hepatocytes in the activation of stellate cells, we incubated HSC 8B cells with EVs from primary cultured hepatocytes (**Figure 7**). First, to examine the incorporation of RNA from primary RH-derived EVs, we used the liver-specific transcript *Alb* coding for Albumin, which is not expressed in 8B cells [38]. The PCR amplification of this transcript could be observed only in 8B cells incubated with intact hepatocyte-derived EVs (**lane 2,** **Figure 7A**). This result strongly suggests a genetic transfer through EVs. Furthermore, we were able to amplify *Alb* transcript up to 24 hours after the incubation with EVs, which indicates that the RNA is not degraded immediately. Next, we evaluated the effect of these RH-derived EVs on the activation of the stellate-like cell line. As previously described [38], functional activation of 8B cells can be assessed by the measurement of intracellular levels of the transcript coding for nitric oxide synthase 2 (*Nos2)*, which is considered a marker of activated HSC [39], [40]. The transcription of *Nos2* in 8B cells was clearly increased by incubation with hepatocyte-derived EVs (**Figure 7B**), confirming stellate-activating properties of these vesicles. Importantly, this effect was partly mediated by the RNA carried by the EVs; pre-treating EVs with RNase in presence of Tx-100 inhibited this process (**Figure 7B**). It is noticeable that pre-treatment of EVs with Tx-100 also reduces the induction on Nos2. This effect may be explained by a reduction in the uptake efficiency, as showed in Figure S3.

### RNA Cargo of Hepatocyte-Derived EVs Constitutes a Source for Diagnostic Purposes

**Figure 8:**
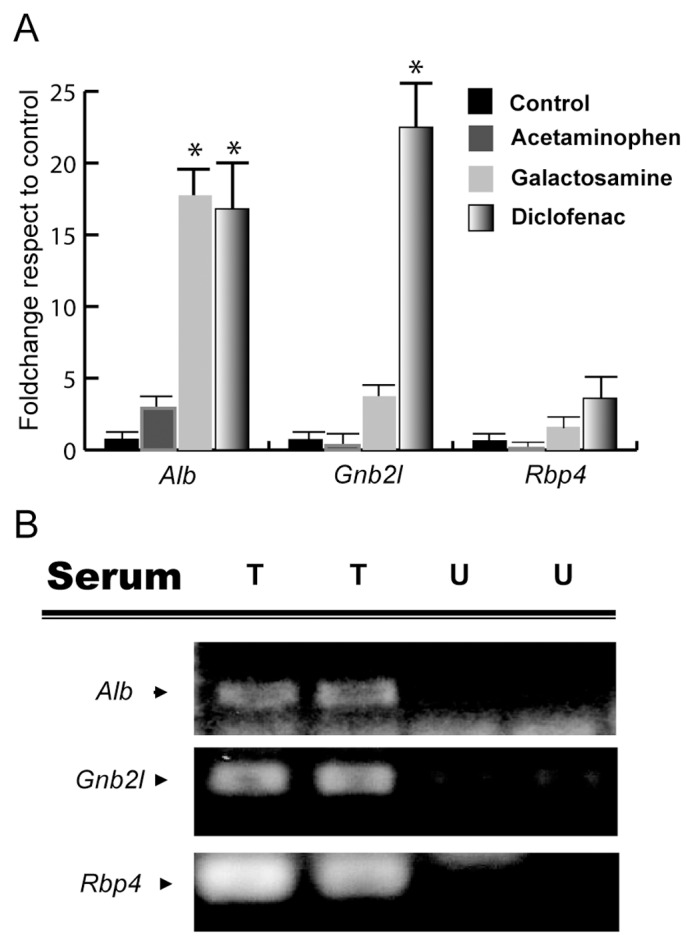
RNA content of hepatic EVs could be useful in hepatotoxicity diagnostics. (A) Q-PCR analysis of the RNA cargo of EVs released by hepatocytes after exposure to indicated drugs. (error bars represent SD (n = 2), * denotes *p*<0.05 with respect to the control). (B) Q-PCR analysis of RNA cargo of EVs isolated from serum of untreated (U) rats and rats treated (T) with galactosamine (inducing acute liver injury).

In recent years, the number of studies examining the possible application of EVs in medical diagnosis has increased greatly [41]. We analyzed by qPCR some transcripts from EVs released from primary culture of hepatocytes treated with hepatotoxic drugs galactosamine [42], acetaminophen [43], and diclofenac [44] (**Figure 8A**). After treatment with these drugs, EVs were purified from the culture media using Exoquick reagent (instead of ultracentrifugation), a method more suitable for clinical practice. As shown in **Figure 8A**, we found remarkable differences between the RNA cargos of EVs released after different treatments. The treatment with liver-damaging drugs clearly increased levels of some RNAs in the culture media, such as *Alb*, *Gnb2l*, and *Rbp4*. In the drug-treated hepatocytes, the amounts of *Alb* transcript increased from 2 to more than 15-fold in comparison with vehicle-treated hepatocytes. The levels of transcripts coding for Gnb2l and Rbp4 proteins only increased significantly after galactosamine and diclofenac treatments; the increase was more pronounced in the latter case (**Figure 8A**). These results suggest that RNA cargo isolated from hepatocyte-released EVs could serve as a source of hepatotoxicity indicators *in vitro*. To complete this study, RNAs obtained from EVs were analyzed as putative indicators of liver injury *in vivo*. For this specific purpose, we purified EVs from serum of rats treated with galactosamine (controls treated with saline solution) using Exoquick reagent (**Figure 8B**). EVs from the same volume of serum from each animal were purified as described in *Materials and Methods*. As shown in **Figure 8B** **,** the amplification of RNAs associated with EVs, such as *Alb*, *Gnb2l* and *Rbp4* was observed only in galactosamine-injured animals, in agreement with the *in vitro* results.

## Discussion

The study of EVs as mediators of physiological and pathological processes [11], therapeutics agents [45], and disease biomarkers [46] has evolved rapidly in the recent years. The complexity of their bioactive cargo, including proteins, RNA, microRNA, and DNA, suggests multiple stages and mechanisms by which these vesicles execute their functions. It is very important to characterize the components of EVs in different cellular systems to understand their functions and to elucidate the role of the RNA cargo in their activities. Early studies have shown that EVs released by mast cells contain their own transcriptome formed by at least 1300 different messenger RNAs [13]. Interestingly, this RNA cargo is modified after exposure to oxidative stress, and the changes affect the ability of recipient cells to handle oxidative stress [47]. RNA cargo has been also characterized in EVs derived from cardiomyocytes; these vesicles contain at least 1520 transcripts. Some of the transcripts encode proteins involved in mitochondrial energy generation, suggesting a metabolic role for these vesicles [33]. In EVs released by endothelial progenitor cells, at least 298 different transcripts were identified, and a role in the activation of angiogenic program in quiescent endothelial cells has been proposed for these vesicles [48]. De Jong and co-workers have reported the presence of at least 1992 different transcripts in EVs released by microvascular endothelial cells; they also demonstrated that the RNA cargo of these vesicles changed under hypoxia or endothelial activating conditions [34]. In EVs released by embryonic [49] or liver [50] stem cells, 27 and 65 different transcripts, respectively, have been detected and categorized. These transcripts are likely to be involved in reprogramming haematopoietic progenitors [49] and acceleration of hepatic regeneration [50]. These studies reveal the complexity of the composition and functions of the RNA cargo of EVs and highlight the need for further research on coding RNAs in EVs released by non-tumoral cells. In the current work, we characterized the coding RNA cargo of EVs released by primary hepatocytes, detecting more than 1300 different transcripts. In agreement with the results reported for mast cells [47], microvascular endothelial [34] cells, and cardiomyocytes, we found a significant enrichment in transcripts related to transcription, response to oxidative stress, and energy production, indicating that EVs could play a general role in these processes independently of their cellular origin. In addition, we detected enrichment in gene transcripts coding for proteins involved in lipid, small molecule and xenobiotic metabolism, which are the functional processes of hepatocytes. We also analyzed the RNA cargo in another non-tumoral hepatocyte-like model, MLP29 cells, obtained from foetal liver and presenting progenitor features [19]. In this model, we detected the presence of more than 6500 different transcripts. We found enrichment for the gene transcripts coding for regulatory proteins belonging to various signalling pathways, such as EIF4, mTOR, and PI3K/AKT, controlling cell cycle, cellular survival, and proliferation. Thus, like in EVs obtained from primary hepatocytes, the RNA cargo of MLP29-derived EVs reflects the cellular origin of the vesicles; this feature could be useful for diagnostics purposes. We also observed that, unlike the established cell lines, primary cultures of hepatocytes release a heterogeneous population of EVs carrying different RNA and protein cargos. This phenomenon highlights the complexity of intercellular communication mediated by EVs in the liver [36]. Maintaining affinity of each target for a different vesicle subpopulation requires complex cell systems to trigger specific responses. With more than a thousand transcripts, either messenger or regulatory non-coding RNAs, it is difficult to predict the possible effect of RNA cargo in the recipient cells. In the present work, we showed that the transcripts are captured and that the acceptor cells respond to the RNA cargo. Previously published results have demonstrated that EVs obtained from human liver stem cells accelerate regeneration of the liver *in vivo* [50] and that this effect is RNA-dependent. Here, we report that the *Nos2* induction in HSC 8B, which has been also observed in other studies [38], is partially dependent of RNA cargo of hepatic EVs. The increase in *Nos2* levels in HSC 8B cells is a marker of cell activation. Nitric oxide synthase 2, the product of *Nos2* gene, is involved in triggering systemic vascular responses associated with fibrotic and cirrhosis processes [39], [40] and also with regeneration [51]. It has been shown that EVs released by liver stem cells accelerate liver regeneration [50]. The activation of HSCs after liver injury has been described as a transition from quiescent cells into a fibrogenic, proliferative and contractile fibroblasts, which are critical in liver regeneration (reviewed in [37], [52]). Other studies have demonstrated the activation of HSC by EVs derived from active HSC and T cells [38], [53]. Acute injury induces the release of EVs carrying haematopoietic stem cell markers [54]; this gives some weight to the hypothesis that to maintain its functionality, the liver interacts with other tissues via circulating EVs [36].

Recently, blood-circulating EVs have become a subject of growing interest as a minimally invasive resource for disease diagnosis and treatment monitoring [46]. However, one of the major challenges is to translate the basic research to the clinical practice; to do that, we must be able to purify circulating EVs from small volumes, using a methodology which can be applied in the field. In our study, we tested the performance of Exoquick reagent in obtaining fractions enriched in EVs (extracted from tissue culture media and blood serum samples), with the purpose of analyzing their RNA content. Our data show that Exoquick reagent cannot precipitate naked RNA, and the RNA purified from culture media using this reagent is resistant to RNase activity (**Figure S4**). These results confirm that Exoquick is suitable for use in the diagnostic procedures designed to analyze EVs-associated RNA. Several studies have proposed characterization of EVs as a source of new disease biomarkers. Brodsky and co-workers have shown that the levels of plasma-circulating EVs in liver transplant patients dynamically change after surgery and correlate with the clinical outcome, providing a marker of the functional status of transplanted liver [55]. Moreover, the levels of circulating liver-specific messenger RNAs for proteins such as albumin, haptoglobin, and fibrinogen B can be also used as indicators of liver injury in rats treated with hepatotoxicants (D-galactosamine and acetaminophen) [56] although vesicular association of those transcripts has not been examined. Our work lends further support to the conclusions of that study; we confirmed that the levels of Albumin transcript are increased in EVs-enriched fractions from *in vitro* and *in vivo* liver injury models. We also demonstrated that, by examining the levels of *Alb* and *Gnb2l* transcripts, it is possible to distinguish between the treatments used in this work: D-galactosamine, acetaminophen, and diclofenac. Albumin levels were increased by all 3 treatments. The levels of *Gnb2l* increased 5- and 20-fold after D-galactosamine and diclofenac treatment, respectively, and acetaminophen treatment had no effect on the levels of this transcript (**Figure 8A**). Overall, these results suggest that some messenger RNAs contained in EVs of hepatic origin could be added to the repertoire of hepatic markers for detection of different liver injuries.

In conclusion, the current work demonstrates the presence of messenger RNAs in EVs released by non-tumoral hepatocytes and shows that those messenger RNAs might play a role in the activation of HSCs, the cells involved in the regulation of liver inflammation and regeneration. Furthermore, this project brings to light the complexity of the content of these vesicles as well as the importance of vesicle cargo characterization in various EVs populations, with functional and diagnostic perspectives in mind.

## Supporting Information

Figure S1 **Confirmation of the RNA identification obtained by array hybridization.** Array intensity trends and obtained qPCR-Cts correlation for a group of genes. Each point is the average of three independent qPCR experiments for different EVs preparations *vs*. the array results for one sample MLP29 (A) and one sample RH (B).
(TIF)

Figure S2 **Integrity of the transcripts loaded in EVs.** Amplification of the whole coding sequence of Anp32b protein was achieved using RNAs from EVs derived from MLP29 and RH cells as templates.
(TIF)

Figure S3 **Treatment with Tx-100 reduces the efficiency of EVs capture.** (A) Stellate-like 8B cells were incubated with fluorescently labelled-EVs derived from primary rat hepatocytes either untreated or RNAse-treated with RNase in presence of Tx-100 previous to the incubation. The capture was visualized after 6 hours by confocal microscopy. To highlight the differences, representative images of the same field with low and high brightness are showed for each condition. (B) 8B cells were incubated in the absence or presence of RH-derived EVs that were pre-treated as indicated. After 6-hours cells were recovered, RNA extracted and subjected of RT-PCR to amplify *Alb* transcript. Consistently with the phenomenon observed in (A), Tx-100 pre-treatment (lane 4), reduce the presence of *Alb* transcript in the recipient 8B cells. Pre-treatment of the EVs with Tx-100 and RNase (lane 5) totally abrogated the detection of *Alb* transcript in 8B cells, as showed in Figure 7.
(TIF)

Figure S4 **PCR amplifications of** ***Net1*** **,** ***hSOD*** **and** ***TRAK2*** **transcripts from samples obtained from cell media using Exoquick.** (A) The transcripts obtained are resistant to exogenous RNase activity in the cell media. (B) Exoquick does not recover exogenous purified human RNA added to the cell culture.
(TIF)

Table S1 **Primers employed for PCR and qPCR amplifications in the study.**
(DOC)

Table S2 **Most abundant transcripts (according to signal intensity of the array) in EVs derived from RH.**
(DOC)

Table S3 **Top 50 enriched transcripts in MLP29 EVs when compared to MLP29 cells.**
(DOC)

Table S4 **Transcripts only detected in MLP29 and RH after comparison with other published EVs transcriptomes.**
(DOC)

Table S5 **Transcripts common to different published list of EVs mRNA, MLP29 and RH.**
(DOC)
