# Supplementary figures and images for: Transcriptome of Extracellular Vesicles Released by Hepatocytes
Source: PLoS One. 2013 Jul 11;8(7):e68693. doi: 10.1371/journal.pone.0068693 (PMC3708910; doi:10.1371/journal.pone.0068693)

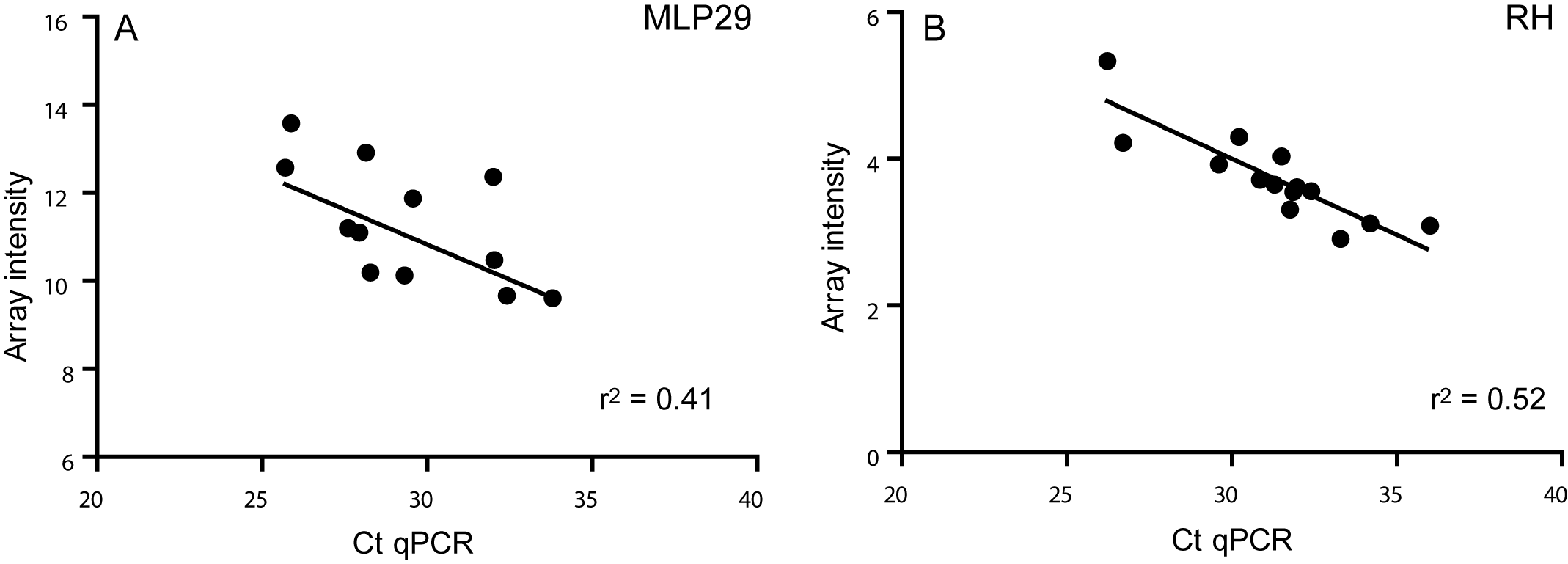

Supplement: Figure S1 — Confirmation of the RNA identification obtained by array hybridization. Array intensity trends and obtained qPCR-Cts correlation for a group of genes. Each point is the average of three independent qPCR experiments for different EVs preparations vs. the array results for one sample MLP29 (A) and one sample RH (B). (TIF) [file pone.0068693.s001.tif]

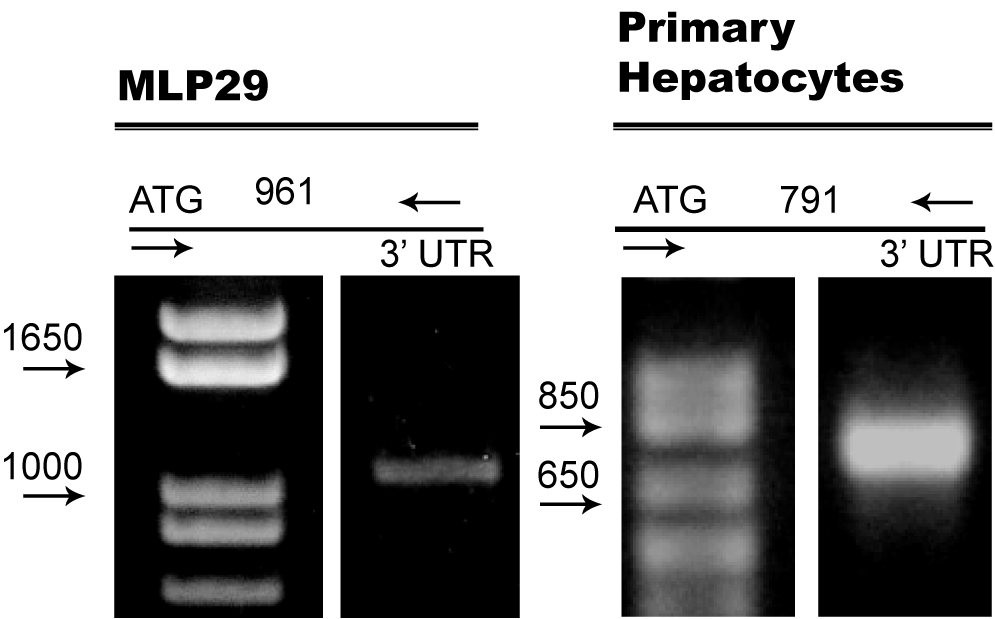

Supplement: Figure S2 — Integrity of the transcripts loaded in EVs. Amplification of the whole coding sequence of Anp32b protein was achieved using RNAs from EVs derived from MLP29 and RH cells as templates. (TIF) [file pone.0068693.s002.tif]

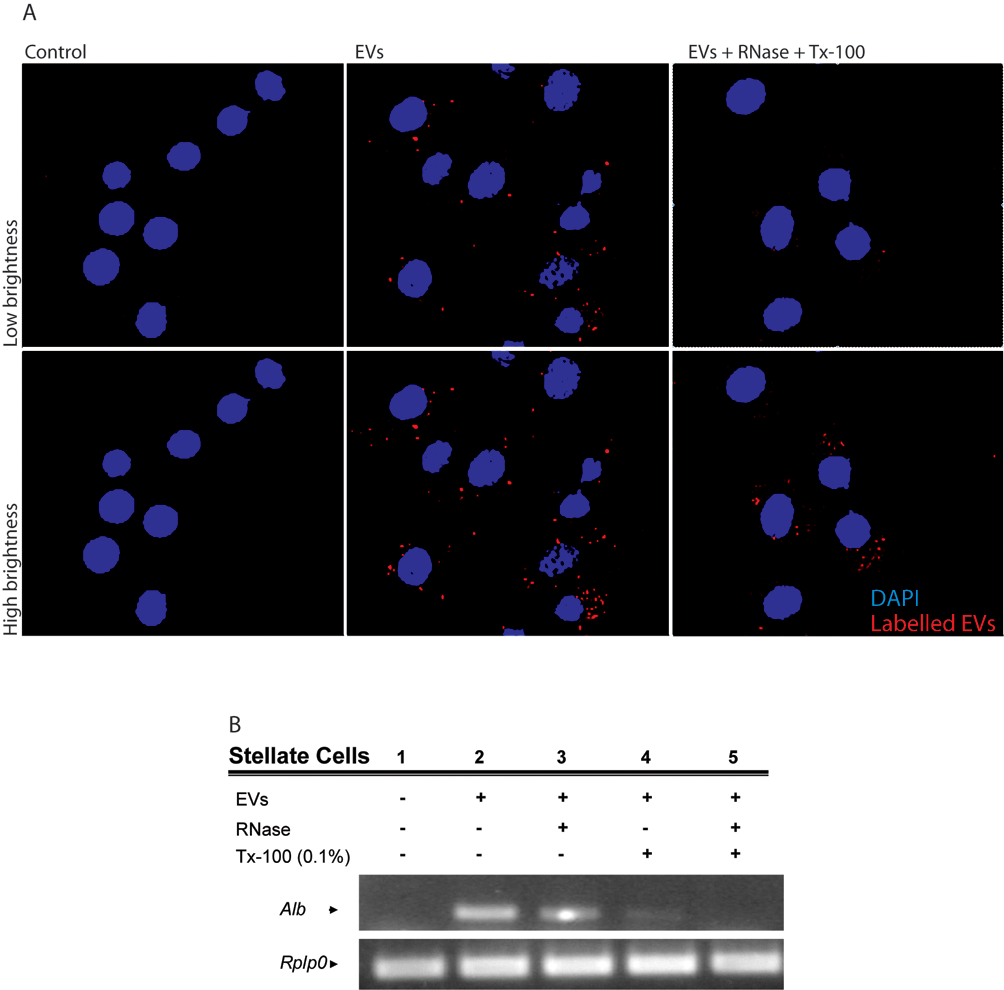

Supplement: Figure S3 — Treatment with Tx-100 reduces the efficiency of EVs capture. (A) Stellate-like 8B cells were incubated with fluorescently labelled-EVs derived from primary rat hepatocytes either untreated or RNAse-treated with RNase in presence of Tx-100 previous to the incubation. The capture was visualized after 6 hours by confocal microscopy. To highlight the differences, representative images of the same field with low and high brightness are showed for each condition. (B) 8B cells were incubated in the absence or presence of RH-derived EVs that were pre-treated as indicated. After 6-hours cells were recovered, RNA extracted and subjected of RT-PCR to amplify Alb transcript. Consistently with the phenomenon observed in (A), Tx-100 pre-treatment (lane 4), reduce the presence of Alb transcript in the recipient 8B cells. Pre-treatment of the EVs with Tx-100 and RNase (lane 5) totally abrogated the detection of Alb transcript in 8B cells, as showed in Figure 7. (TIF) [file pone.0068693.s003.tif]

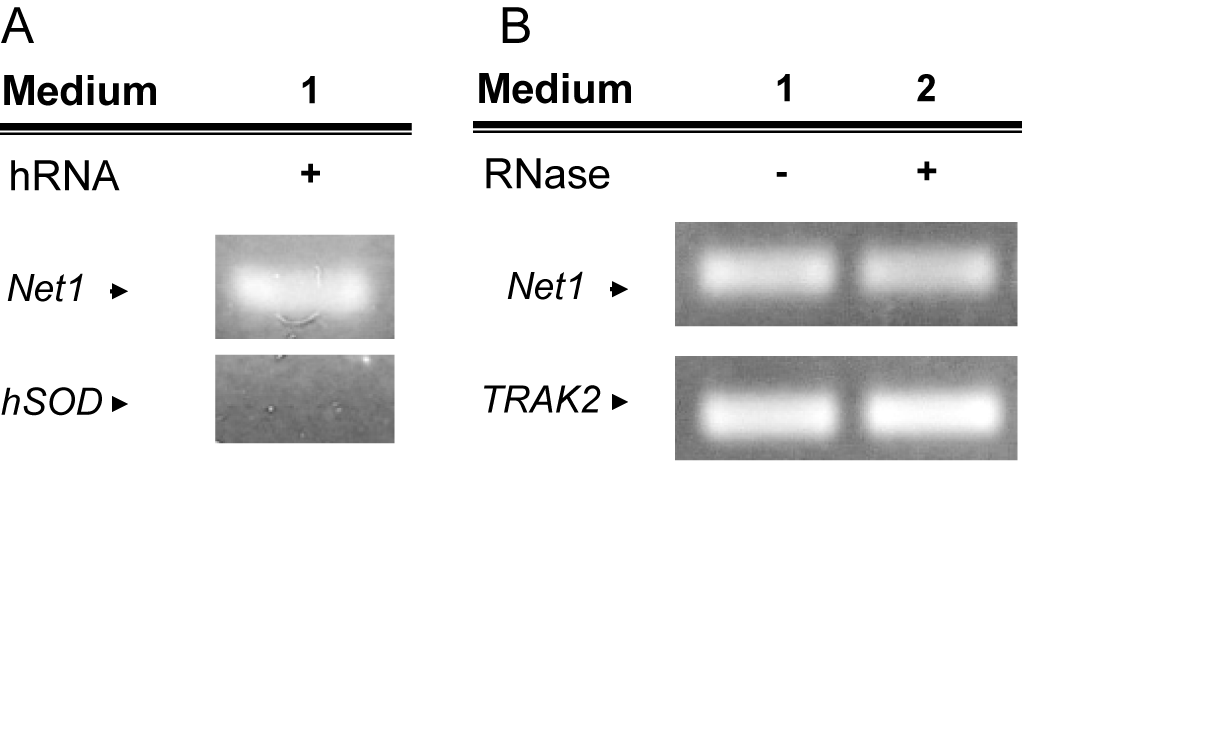

Supplement: Figure S4 — PCR amplifications of Net1 , hSOD and TRAK2 transcripts from samples obtained from cell media using Exoquick. (A) The transcripts obtained are resistant to exogenous RNase activity in the cell media. (B) Exoquick does not recover exogenous purified human RNA added to the cell culture. (TIF) [file pone.0068693.s004.tif]
