# Supplementary material for: Transcriptome of Extracellular Vesicles Released by Hepatocytes
Source: PLoS One. 2013 Jul 11;8(7):e68693. doi: 10.1371/journal.pone.0068693 (PMC3708910; doi:10.1371/journal.pone.0068693)
Supplement: Table S1 — Primers employed for PCR and qPCR amplifications in the study. (DOC) [file pone.0068693.s005.doc]

**Table S1**. **Primers employed for PCR and qPCR amplifications in the study.**

| **Gene** | **Species** | **Forward** | **Reverse** | **Designed** | **Size** | **Validation** |
| --- | --- | --- | --- | --- | --- | --- |
| **Anp32b** | Mouse | GACCAGGAAGCACCTGACTC | CGCTGACTTCATCCTCATCA | Primer3 | 223 | F 3B |
| **Arf1** | Mouse | GCTGCAGGGAAGACAACAAT | ATCAAGCCTTGGGTGTTCTG | Primer3 | 188 | F 3B |
| **Cd81** | Mouse | TTCCATGAGACGCTCAACTG | TGAGGTACAGCTTCCCAGAGA | Primer3 | 166 | F 3B |
| **Ei24** | Mouse | GGGTCAGCTGGTTAGTCTGC | TTGCATTGCTGTGAGGAAAG | Primer3 | 172 | F 3B- SF 1A |
| **Gapdh** | Mouse | AACGACCCCTTCATTGAC | TCCACGACATACTCAGCAC | (1) | 191 | F 3B- SF 1A |
| **MAPK9** | Mouse | GATCCAGACAAGCGGATCTC | GGCTGGTCTTTTACCCCATT | Primer3 | 212 | F 3B- SF 1A |
| **Mif** | Mouse | CAAGCCCGCACAGTACATC | AGGCCACACAGCAGCTTACT | Primer3 | 153 | F 3B- SF 1A |
| **Net1** | Mouse | CGGCAACTCAGACAAAGCTA | CCAGCAGAAGGGTTGTTCTC | Primer3 | 222 | F 3B- SF 1A |
| **Nme2** | Mouse | GATTCAAAACCAGGCACCAT | GGGCTTAAACCACAGATGGA | Primer3 | 117 | F 3B- SF 1A |
| **Rassf3** | Mouse | CCACCAAGTTTGCGCTTTAT | CCCACTCTCCAATTTCATGC | Primer3 | 159 | F 3B- SF 1A |
| **Rps9** | Mouse | ATCCCATCCTTCATTGTTCG | TCTTGGCATTCTTCCTCTTCA | Primer3 | 109 | F 3B- SF 1A |
| **Smad4** | Mouse | AGCTCCAGCCATCAGTCTGT | GCAGGACTTCATCCAAGAGC | Primer3 | 188 | F 3B- SF 1A |
| **Tgfb1** | Mouse | GTGGAAATCAACGGGATCAG | CGCACACAGCAGTTCTTCTC | Primer3 | 188 | F 3B- SF 1A |
| **Trak2** | Mouse | TTCGAACCCAGGAAAGTGTC | GCTCCTGGTGCTACTGAAGG | Primer3 | 250 | F 3B- SF 1A |
| **Vegf1b** | Mouse | CAGCCAATGTGAATGCAGAC | GGAGTGGGATGGATGATGTC | Primer3 | 153 | F 3B- SF 1A |
| **Alb** | Rat | AGCAGCCTGCCTGACACCGA | CAGGTCGCCGTGACAGCACT | Primer3 | 229 | SF 1B |
| **Anp32b** | Rat | TCGGAGGTCTCGACAGACTAGCAG | ACGGTCTCTCGGTAATCACTCCG | Primer3 | 175 | SF 1B |
| **Apoc1** | Rat | GCTTTGGAAGGCCCAGCCCC | TGGCTGCCCGGGCCTTATCT | Primer3 | 118 | SF 1B |
| **Cyp1a2** | Rat | CCCAGGAAGAGCGAGGAGATGCT | GGGTTGGGCAGGTAGCGCAG | Primer3 | 116 | SF 1B |
| **Cyp2e1** | Rat | TGGAACCTGCCCCCAGGACC | ACCACGATGCGCCTTGAGCC | Primer3 | 149 | SF 1B |
| **Ei24** | Rat | TCAGTGGCTGCCTCTTTTCT | CTGCAGCAGCTTTCAGTTTG | Primer3 | 208 | SF 1B |
| **Fabp1** | Rat | CGGCAAGTACCAAGTGCAGAGCC | CTCCAGTTCGCACTCCTCCCCC | Primer3 | 205 | SF 1B |
| **Gnb2l** | Rat | CCACTCCGCAGTTCCCGGAC | TCGTCTCGTGGTAGTGCCCGT | Primer3 | 233 | SF 1B |
| **Igf1** | Rat | TTTGCGGGGCTGAGCTGGTG | ATGTCAGTGTGGCGCTGGGC | Primer3 | 229 | SF 1B |
| **MSGT** | Rat | TGTGCTGGCTTCGGCAAGGG | ATCCGGTCCGCTCAGGGAGT | Primer3 | 150 | SF 1B |
| **Rassf3** | Rat | AGCAGGGCCCAGAACCGACA | TCATGCGTCCCGAGCTGTGC | Primer3 | 247 | SF 1B |
| **Rpl27** | Rat | GGTCAGCGAGCAGACACGGG | GCCACCAGGACACAGGCACC | Primer3 | 201 | SF 1B |
| **Rarres2** | Rat | AGCTGGCACCTTTGTGAGGCTG | CCTCGCCGGCCTGTGCTATC | Primer3 | 242 | SF 1B |
| **Ugt1A6 (and others)** | Rat | ACTCGGGGAGCTGGGGTGAC | GCGCCCCCTTGTGCCTCATC | Primer3 | 199 | SF 1B |
| **Cyp2d1** | Rat | GCAGGTGGACCTCAGTAACATGC | CTCTCGCCACTCAGGCCCGT | Primer3 | 250 |  |
| **Rplp0** | Mouse | CGACCTGGAAGTCCAACTAC | ATCTGCTGCATCTGCTTG | (1) | 109 |  |
| **Nos2** | Rat | CTGAGACTCTGGCCCCACGGG | ACTTCCAGGGGCAAGCCATGTCT | Primer3 | 200 |  |
| **hSOD** | Human | TCGCCCAATAAACATTCCCTTG | AAGTCTGGCAAAATACAGGTCATTG | (2) | 249 |  |
| **Anp32b** | Mouse | ATGGATATGAAGAGGAGGATCC | CCAATCACAGCTGTCACGAT | Primer3 | 961 | Integrity |
| **Anp32b** | Rat | ATGGACATGAAGAGGAGGAT | TCTCTCCTCTGTCACCACCA | Primer3 | 791 | Integrity |

1. Simpson DA, Feeney S, Boyle C, Stitt AW. Retinal VEGF mRNA measured by SYBR green I fluorescence: A versatile approach to quantitative PCR. Mol Vis. 2000;6:178-83. Epub 2000/10/07.

2. Ait Yahya-Graison E, Aubert J, Dauphinot L, Rivals I, Prieur M, Golfier G, et al. Classification of human chromosome 21 gene-expression variations in Down syndrome: impact on disease phenotypes. Am J Hum Genet. 2007;81(3):475-91. Epub 2007/08/1
