# Supplementary material for: Transcriptome of Extracellular Vesicles Released by Hepatocytes
Source: PLoS One. 2013 Jul 11;8(7):e68693. doi: 10.1371/journal.pone.0068693 (PMC3708910; doi:10.1371/journal.pone.0068693)
Supplement: Table S2 — Most abundant transcripts (according to signal intensity of the array) in EVs derived from RH. (DOC) [file pone.0068693.s006.doc]

**Table S2**. Most abundant transcripts (according to signal intensity of the array) in EVs derived from RH

| Symbol | Entrez Gene Name | Location | Type(s) | Intensity |
| --- | --- | --- | --- | --- |
| ALB* | albumin | Extracellular Space | transporter | 214137 |
| Rup2 | urinary protein 2 | unknown | other | 111988 |
| COX1 (includes EG:140539) | cytochrome c oxidase subunit I | Cytoplasm | enzyme | 98669 |
| LOC257642 | rRNA promoter binding protein | Nucleus | transcription regulator | 66042 |
| ND1 (includes EG:140531) | NADH dehydrogenase, subunit 1 (complex I) | Cytoplasm | enzyme | 65450 |
| CYTB (includes EG:140512) | cytochrome b | Cytoplasm | enzyme | 57777 |
| ND2 (includes EG:140532) | MTND2 | Cytoplasm | enzyme | 52592 |
| COX2 (includes EG:140540) | cytochrome c oxidase subunit II | Cytoplasm | enzyme | 46004 |
| TTR | transthyretin | Extracellular Space | transporter | 40923 |
| TF | transferrin | Extracellular Space | transporter | 39487 |
| APOC3 | apolipoprotein C-III | Extracellular Space | transporter | 33694 |
| ND3 (includes EG:140533) | NADH dehydrogenase, subunit 3 (complex I) | Cytoplasm | enzyme | 31683 |
| SERPINA1 | serpin peptidase inhibitor, clade A (alpha-1 antiproteinase, antitrypsin), member 1 | Extracellular Space | other | 30051 |
| HP | haptoglobin | Extracellular Space | peptidase | 21798 |
| FTH1 (includes EG:14319) | ferritin, heavy polypeptide 1 | Cytoplasm | enzyme | 19823 |
| MGST1* | microsomal glutathione S-transferase 1 | Cytoplasm | enzyme | 19752 |
| GC | group-specific component (vitamin D binding protein) | Extracellular Space | transporter | 18975 |
| APOC1* | apolipoprotein C-I | Extracellular Space | transporter | 16493 |
| RBP4 | retinol binding protein 4, plasma | Extracellular Space | transporter | 12633 |
| AHSG | alpha-2-HS-glycoprotein | Extracellular Space | other | 12556 |
| RPS27* | ribosomal protein S27 | Cytoplasm | other | 11770 |
| RPS19 | ribosomal protein S19 | Cytoplasm | other | 10898 |
| FABP1* | fatty acid binding protein 1, liver | Cytoplasm | transporter | 10801 |
| APOH | apolipoprotein H (beta-2-glycoprotein I) | Extracellular Space | transporter | 10595 |
| RPL41 | ribosomal protein L41 | Cytoplasm | other | 10508 |
| CA3 | carbonic anhydrase III, muscle specific | Cytoplasm | enzyme | 10489 |
| RPL23 | ribosomal protein L23 | Cytoplasm | other | 10152 |
| RPS14 | ribosomal protein S14 | Cytoplasm | translation regulator | 10043 |
| TMSB10/TMSB4X | thymosin beta 4, X-linked | Cytoplasm | other | 9719 |
| APOE | apolipoprotein E | Extracellular Space | transporter | 9623 |
| RPS29 | ribosomal protein S29 | Cytoplasm | other | 9529 |
| RPS2 | ribosomal protein S2 | Cytoplasm | other | 9453 |
| TPT1 (includes EG:100043703) | tumor protein, translationally-controlled 1 | Cytoplasm | other | 9233 |
| RPS18 | ribosomal protein S18 | Cytoplasm | other | 8619 |
| RPS23 | ribosomal protein S23 | Cytoplasm | translation regulator | 8592 |
| RPL27A | ribosomal protein L27a | Nucleus | other | 8408 |
| ACTB | actin, beta | Cytoplasm | other | 8293 |
| Gm6402 | ribosomal protein S17 pseudogene | Cytoplasm | other | 8123 |
| AMBP | alpha-1-microglobulin/bikunin precursor | Extracellular Space | transporter | 8113 |
| Rpl9 | ribosomal protein L9 | Nucleus | other | 8059 |
| APOA2 | apolipoprotein A-II | Extracellular Space | transporter | 7758 |
| B2M | beta-2-microglobulin | Plasma Membrane | transmembrane receptor | 7723 |
| RPS8 | ribosomal protein S8 | Cytoplasm | other | 7700 |
| RPL26 | ribosomal protein L26 | Cytoplasm | other | 7615 |
| SEPP1 | selenoprotein P, plasma, 1 | Extracellular Space | other | 7505 |
| LOC299282 | Serine protease inhibitor | Extracellular Space | other | 7444 |
| FTL | ferritin, light polypeptide | Cytoplasm | enzyme | 7434 |
| Eef1a1 | eukaryotic translation elongation factor 1 alpha 1 | Cytoplasm | translation regulator | 7275 |
| RPS12 | ribosomal protein S12 | Cytoplasm | other | 7064 |

* Genes employed in the validation of Supplemental Figure 1B
