# Supplementary material for: Transcriptome of Extracellular Vesicles Released by Hepatocytes
Source: PLoS One. 2013 Jul 11;8(7):e68693. doi: 10.1371/journal.pone.0068693 (PMC3708910; doi:10.1371/journal.pone.0068693)
Supplement: Table S3 — Top 50 enriched transcripts in MLP29 EVs when compared to MLP29 cells. (DOC) [file pone.0068693.s007.doc]

**Table S3. Top 50 enriched transcripts in MLP29 EVs when compared to MLP29 cells.**

| Symbol | Entrez Gene Name | Location | Type(s) | Fold Change |
| --- | --- | --- | --- | --- |
| CST6 (includes EG:1474) | cystatin E/M | Extracellular Space | other | -194.707 |
| TRAK2* | trafficking protein, kinesin binding 2 | Plasma Membrane | transporter | -80.473 |
| PKP4 | plakophilin 4 | Plasma Membrane | other | -67.434 |
| KIF1C | kinesin family member 1C | Cytoplasm | other | -64.678 |
| NET1* (includes EG:10276) | neuroepithelial cell transforming 1 | Nucleus | other | -45.608 |
| PKP4 | plakophilin 4 | Plasma Membrane | other | -41.758 |
| DNAJC30 | DnaJ (Hsp40) homolog, subfamily C, member 30 | Cytoplasm | other | -35.705 |
| CHAF1A | chromatin assembly factor 1, subunit A (p150) | Nucleus | other | -30.070 |
| RASSF3* | Ras association (RalGDS/AF-6) domain family member 3 | unknown | other | -27.391 |
| PKP4 | plakophilin 4 | Plasma Membrane | other | -26.719 |
| Gm10125 | predicted gene 10125 | unknown | other | -26.226 |
| PRAMEF13 (includes others) | PRAME family member 13 | unknown | other | -25.964 |
| SLC11A1 | solute carrier family 11 (proton-coupled divalent metal ion transporters), member 1 | Plasma Membrane | transporter | -24.899 |
| ANP32B* | acidic (leucine-rich) nuclear phosphoprotein 32 family, member B | Nucleus | other |  |
| ZEB1 | zinc finger E-box binding homeobox 1 | Nucleus | transcription regulator | -24.014 |
| ACTB | actin, beta | Cytoplasm | other | -23.970 |
| C1orf115 | chromosome 1 open reading frame 115 | unknown | other | -23.156 |
| KCTD10 | potassium channel tetramerisation domain containing 10 | Nucleus | ion channel | -21.860 |
| HSD3B1 | hydroxy-delta-5-steroid dehydrogenase, 3 beta- and steroid delta-isomerase 1 | Cytoplasm | enzyme | -21.842 |
| FCHSD1 | FCH and double SH3 domains 1 | unknown | other | -21.638 |
| TP53INP2 | tumor protein p53 inducible nuclear protein 2 | Nucleus | other | -20.353 |
| CYB5R3 | cytochrome b5 reductase 3 | Cytoplasm | enzyme | -19.589 |
| Ccdc144b | coiled-coil domain containing 144B | unknown | other | -19.476 |
| KIF4A | kinesin family member 4A | Nucleus | other | -18.563 |
| UACA | uveal autoantigen with coiled-coil domains and ankyrin repeats | Cytoplasm | other | -18.419 |
| Gm9753 | predicted gene 9753 | unknown | other | -18.336 |
| MAP2K7 | mitogen-activated protein kinase kinase 7 | Cytoplasm | kinase | -17.996 |
| NIN | ninein (GSK3B interacting protein) | Cytoplasm | other | -17.799 |
| GXYLT1 | glucoside xylosyltransferase 1 | unknown | other | -17.622 |
| MAPKBP1 | mitogen-activated protein kinase binding protein 1 | unknown | other | -16.941 |
| Ncl | nucleolin | Nucleus | other | -16.369 |
| CGNL1 | cingulin-like 1 | Plasma Membrane | other | -15.759 |
| TERF2IP | telomeric repeat binding factor 2, interacting protein | Nucleus | other | -15.127 |
| SPC24 (includes EG:147841) | SPC24, NDC80 kinetochore complex component, homolog (S. cerevisiae) | Cytoplasm | other | -14.533 |
| ZFPM2 | zinc finger protein, multitype 2 | Nucleus | transcription regulator | -14.428 |
| CGNL1 | cingulin-like 1 | Plasma Membrane | other | -13.903 |
| HTATSF1 | HIV-1 Tat specific factor 1 | Nucleus | transcription regulator | -13.730 |
| KANK2 | KN motif and ankyrin repeat domains 2 | Nucleus | transcription regulator | -13.215 |
| ARNT | aryl hydrocarbon receptor nuclear translocator | Nucleus | transcription regulator | -12.984 |
| CENPB | centromere protein B, 80kDa | Nucleus | other | -12.542 |
| CPLX2 | complexin 2 | Cytoplasm | other | -11.931 |
| FAM40A | family with sequence similarity 40, member A | Nucleus | other | -11.877 |
| Zfp60 (includes others) | zinc finger protein 60 | Nucleus | other | -11.831 |
| KIAA0232 | KIAA0232 | unknown | other | -11.706 |
| PPL | periplakin | Cytoplasm | other | -11.569 |
| CACNB4 | calcium channel, voltage-dependent, beta 4 subunit | Plasma Membrane | ion channel | -11.382 |
| SMPX | small muscle protein, X-linked | Cytoplasm | other | -11.185 |
| CHKA | choline kinase alpha | Cytoplasm | kinase | -11.157 |
| ATPAF2 | ATP synthase mitochondrial F1 complex assembly factor 2 | Cytoplasm | other | -11.154 |

* Genes employed in the validation of Figure 4 and Supplemental Figure 1A
