# Supplementary material for: Transcriptome of Extracellular Vesicles Released by Hepatocytes
Source: PLoS One. 2013 Jul 11;8(7):e68693. doi: 10.1371/journal.pone.0068693 (PMC3708910; doi:10.1371/journal.pone.0068693)
Supplement: Table S4 — Transcripts only detected in MLP29 and RH after comparison with other published EVs transcriptomes. (DOC) [file pone.0068693.s008.doc]

**Table S4. Transcripts only detected in MLP29 and RH after comparison with other published EVs transcriptomes**

| **SYMBOL** | **DEFINITION** |
| --- | --- |
| **ABHD14B** | abhydrolase domain containing 14B (ABHD14B), mRNA. |
| **ABHD2** | abhydrolase domain containing 2 (ABHD2), transcript variant 2, mRNA. |
| **ACAA1** | acetyl-Coenzyme A acyltransferase 1 (peroxisomal 3-oxoacyl-Coenzyme A thiolase) (ACAA1), nuclear gene encoding mitochondrial protein, mRNA. |
| **ACAA2** | acetyl-Coenzyme A acyltransferase 2 (mitochondrial 3-oxoacyl-Coenzyme A thiolase) (ACAA2), nuclear gene encoding mitochondrial protein, mRNA. |
| **ACADL** | acyl-Coenzyme A dehydrogenase, long chain (ACADL), nuclear gene encoding mitochondrial protein, mRNA. |
| **ACSL1** | acyl-CoA synthetase long-chain family member 1 (ACSL1), mRNA. |
| **ACSL5** | acyl-CoA synthetase long-chain family member 5 (ACSL5), transcript variant 2, mRNA. |
| **ADFP** |  |
| **ADSS** | adenylosuccinate synthase (ADSS), mRNA. |
| **AGL** | amylo-1, 6-glucosidase, 4-alpha-glucanotransferase (glycogen debranching enzyme, glycogen storage disease type III) (AGL), transcript variant 6, mRNA. |
| **AHSG** | alpha-2-HS-glycoprotein (AHSG), mRNA. |
| **ALDH2** | aldehyde dehydrogenase 2 family (mitochondrial) (ALDH2), nuclear gene encoding mitochondrial protein, mRNA. |
| **ALDH3A2** | aldehyde dehydrogenase 3 family, member A2 (ALDH3A2), transcript variant 1, mRNA. |
| **ALG9** | asparagine-linked glycosylation 9, alpha-1,2-mannosyltransferase homolog (S. cerevisiae) (ALG9), transcript variant 4, mRNA. |
| **ANP32A** | acidic (leucine-rich) nuclear phosphoprotein 32 family, member A (ANP32A), mRNA. |
| **APG3L** |  |
| **APIP** | APAF1 interacting protein (APIP), mRNA. |
| **APOC2** | apolipoprotein C-II (APOC2), mRNA. |
| **ARAF** | v-raf murine sarcoma 3611 viral oncogene homolog (ARAF), mRNA. |
| **ARBP** |  |
| **ARPP19** | cAMP-regulated phosphoprotein, 19kDa (ARPP19), mRNA. |
| **ATF5** | activating transcription factor 5 (ATF5), mRNA. |
| **ATF7IP** | activating transcription factor 7 interacting protein (ATF7IP), mRNA. |
| **ATP6V0A2** | ATPase, H+ transporting, lysosomal V0 subunit a2 (ATP6V0A2), mRNA. |
| **ATP6V1B2** | ATPase, H+ transporting, lysosomal 56/58kDa, V1 subunit B2 (ATP6V1B2), mRNA. |
| **BACE1** | beta-site APP-cleaving enzyme 1 (BACE1), transcript variant b, mRNA. |
| **BCL6** | B-cell CLL/lymphoma 6 (zinc finger protein 51) (BCL6), transcript variant 2, mRNA. |
| **BRD1** | bromodomain containing 1 (BRD1), mRNA. |
| **C2** | complement component 2 (C2), mRNA. |
| **C3** | complement component 3 (C3), mRNA. |
| **C9** | complement component 9 (C9), mRNA. |
| **CAB39L** | calcium binding protein 39-like (CAB39L), transcript variant 2, mRNA. |
| **CALR** | calreticulin (CALR), mRNA. |
| **CAPN7** | calpain 7 (CAPN7), mRNA. |
| **CCL9** |  |
| **CD164** | CD164 molecule, sialomucin (CD164), mRNA. |
| **CD2BP2** | CD2 (cytoplasmic tail) binding protein 2 (CD2BP2), mRNA. |
| **CDC2L5** | cell division cycle 2-like 5 (cholinesterase-related cell division controller) (CDC2L5), transcript variant 2, mRNA. |
| **CDC37L1** | cell division cycle 37 homolog (S. cerevisiae)-like 1 (CDC37L1), mRNA. |
| **CDK6** | cyclin-dependent kinase 6 (CDK6), mRNA. |
| **CHPT1** | choline phosphotransferase 1 (CHPT1), mRNA. |
| **CITED2** | Cbp/p300-interacting transactivator, with Glu/Asp-rich carboxy-terminal domain, 2 (CITED2), transcript variant 1, mRNA. |
| **CLU** | clusterin (CLU), transcript variant 2, mRNA. |
| **CMPK** |  |
| **CNIH** | cornichon homolog (Drosophila) (CNIH), transcript variant 2, mRNA. |
| **COMMD8** | COMM domain containing 8 (COMMD8), mRNA. |
| **CREM** | cAMP responsive element modulator (CREM), transcript variant 2, mRNA. |
| **CSNK2A2** | casein kinase 2, alpha prime polypeptide (CSNK2A2), mRNA. |
| **CTE1** |  |
| **CTNNB1** | PREDICTED: catenin (cadherin-associated protein), beta 1, 88kDa (CTNNB1), mRNA. |
| **CYB5** |  |
| **CYP20A1** | cytochrome P450, family 20, subfamily A, polypeptide 1 (CYP20A1), transcript variant 1, mRNA. |
| **DAP** | death-associated protein (DAP), mRNA. |
| **DDEF2** | development and differentiation enhancing factor 2 (DDEF2), mRNA. |
| **DDR2** | discoidin domain receptor tyrosine kinase 2 (DDR2), transcript variant 1, mRNA. |
| **DGAT2** | diacylglycerol O-acyltransferase homolog 2 (mouse) (DGAT2), mRNA. |
| **DNAJA2** | DnaJ (Hsp40) homolog, subfamily A, member 2 (DNAJA2), mRNA. |
| **DNCL2A** | dynein, cytoplasmic, light polypeptide 2A (DNCL2A), transcript variant 3, mRNA. |
| **DNMT1** | DNA (cytosine-5-)-methyltransferase 1 (DNMT1), mRNA. |
| **DUSP18** | dual specificity phosphatase 18 (DUSP18), mRNA. |
| **ECH1** | enoyl Coenzyme A hydratase 1, peroxisomal (ECH1), mRNA. |
| **EIF3S3** |  |
| **EIF3S5** |  |
| **EIF3S7** |  |
| **ELOVL5** | ELOVL family member 5, elongation of long chain fatty acids (FEN1/Elo2, SUR4/Elo3-like, yeast) (ELOVL5), mRNA. |
| **EPC2** | enhancer of polycomb homolog 2 (Drosophila) (EPC2), mRNA. |
| **EPRS** | glutamyl-prolyl-tRNA synthetase (EPRS), mRNA. |
| **FBLN2** | fibulin 2 (FBLN2), transcript variant 1, mRNA. |
| **FDXR** | ferredoxin reductase (FDXR), nuclear gene encoding mitochondrial protein, transcript variant 1, mRNA. |
| **FGD3** | FYVE, RhoGEF and PH domain containing 3 (FGD3), transcript variant 2, mRNA. |
| **FH1** |  |
| **FKBP2** | FK506 binding protein 2, 13kDa (FKBP2), transcript variant 1, mRNA. |
| **FKBP5** | FK506 binding protein 5 (FKBP5), mRNA. |
| **FNDC3B** | fibronectin type III domain containing 3B (FNDC3B), transcript variant 1, mRNA. |
| **FOXP1** | forkhead box P1 (FOXP1), transcript variant 1, mRNA. |
| **FTSJ3** | FtsJ homolog 3 (E. coli) (FTSJ3), mRNA. |
| **GABARAP** | GABA(A) receptor-associated protein (GABARAP), mRNA. |
| **GABPB1** | GA binding protein transcription factor, beta subunit 1 (GABPB1), transcript variant gamma-1, mRNA. |
| **GADD45A** | growth arrest and DNA-damage-inducible, alpha (GADD45A), mRNA. |
| **GCDH** | glutaryl-Coenzyme A dehydrogenase (GCDH), nuclear gene encoding mitochondrial protein, transcript variant 2, mRNA. |
| **GCNT1** | glucosaminyl (N-acetyl) transferase 1, core 2 (beta-1,6-N-acetylglucosaminyltransferase) (GCNT1), transcript variant 4, mRNA. |
| **GLUD1** | glutamate dehydrogenase 1 (GLUD1), mRNA. |
| **GNA12** | guanine nucleotide binding protein (G protein) alpha 12 (GNA12), mRNA. |
| **GNAO** |  |
| **GPLD1** | glycosylphosphatidylinositol specific phospholipase D1 (GPLD1), transcript variant 1, mRNA. |
| **GPRK6** |  |
| **GRN** | granulin (GRN), mRNA. |
| **GRSF1** | G-rich RNA sequence binding factor 1 (GRSF1), transcript variant 2, mRNA. |
| **GSTA2** | glutathione S-transferase A2 (GSTA2), mRNA. |
| **GSTA4** | glutathione S-transferase A4 (GSTA4), mRNA. |
| **GTF3C5** | general transcription factor IIIC, polypeptide 5, 63kDa (GTF3C5), mRNA. |
| **HADH2** | hydroxyacyl-Coenzyme A dehydrogenase, type II (HADH2), transcript variant 1, mRNA. |
| **HGFAC** | HGF activator (HGFAC), mRNA. |
| **HIBADH** | 3-hydroxyisobutyrate dehydrogenase (HIBADH), mRNA. |
| **HIP2** | huntingtin interacting protein 2 (HIP2), mRNA. |
| **HIST1H2BH** | histone cluster 1, H2bh (HIST1H2BH), mRNA. |
| **HMGCS1** | 3-hydroxy-3-methylglutaryl-Coenzyme A synthase 1 (soluble) (HMGCS1), transcript variant 2, mRNA. |
| **HNRPH1** | heterogeneous nuclear ribonucleoprotein H1 (H) (HNRPH1), mRNA. |
| **HPRT** |  |
| **HRG** | histidine-rich glycoprotein (HRG), mRNA. |
| **HRSP12** | heat-responsive protein 12 (HRSP12), mRNA. |
| **HSPCA** |  |
| **HSPCB** |  |
| **IDH1** | isocitrate dehydrogenase 1 (NADP+), soluble (IDH1), mRNA. |
| **IGF2R** | insulin-like growth factor 2 receptor (IGF2R), mRNA. |
| **IL24** | interleukin 24 (IL24), transcript variant 2, mRNA. |
| **INCENP** | inner centromere protein antigens 135/155kDa (INCENP), mRNA. |
| **JMJD3** |  |
| **KCTD1** | potassium channel tetramerisation domain containing 1 (KCTD1), mRNA. |
| **KEAP1** | kelch-like ECH-associated protein 1 (KEAP1), transcript variant 2, mRNA. |
| **KNS2** |  |
| **KPNA1** | karyopherin alpha 1 (importin alpha 5) (KPNA1), mRNA. |
| **KRT1-18** |  |
| **KRT2-8** |  |
| **L3MBTL3** | l(3)mbt-like 3 (Drosophila) (L3MBTL3), transcript variant 1, mRNA. |
| **LANCL2** | LanC lantibiotic synthetase component C-like 2 (bacterial) (LANCL2), mRNA. |
| **LITAF** | lipopolysaccharide-induced TNF factor (LITAF), mRNA. |
| **LRRC59** | leucine rich repeat containing 59 (LRRC59), mRNA. |
| **LYPLA1** | lysophospholipase I (LYPLA1), mRNA. |
| **LZTR1** | leucine-zipper-like transcription regulator 1 (LZTR1), mRNA. |
| **MBD3** | methyl-CpG binding domain protein 3 (MBD3), mRNA. |
| **MCFD2** | multiple coagulation factor deficiency 2 (MCFD2), mRNA. |
| **MCL1** | myeloid cell leukemia sequence 1 (BCL2-related) (MCL1), transcript variant 1, mRNA. |
| **MGST1** | microsomal glutathione S-transferase 1 (MGST1), transcript variant 1d, mRNA. |
| **MGST2** | microsomal glutathione S-transferase 2 (MGST2), mRNA. |
| **MINA** | MYC induced nuclear antigen (MINA), transcript variant 3, mRNA. |
| **MLF2** | myeloid leukemia factor 2 (MLF2), mRNA. |
| **MRPL50** | mitochondrial ribosomal protein L50 (MRPL50), nuclear gene encoding mitochondrial protein, mRNA. |
| **MTHFD1** | methylenetetrahydrofolate dehydrogenase (NADP+ dependent) 1, methenyltetrahydrofolate cyclohydrolase, formyltetrahydrofolate synthetase (MTHFD1), mRNA. |
| **MVP** | major vault protein (MVP), transcript variant 1, mRNA. |
| **NEU1** | sialidase 1 (lysosomal sialidase) (NEU1), mRNA. |
| **NIT1** | nitrilase 1 (NIT1), mRNA. |
| **NSBP1** | nucleosomal binding protein 1 (NSBP1), mRNA. |
| **NSDHL** | NAD(P) dependent steroid dehydrogenase-like (NSDHL), mRNA. |
| **NSUN2** | NOP2/Sun domain family, member 2 (NSUN2), mRNA. |
| **NUDT4** | nudix (nucleoside diphosphate linked moiety X)-type motif 4 (NUDT4), transcript variant 1, mRNA. |
| **NUPR1** | nuclear protein, transcriptional regulator, 1 (NUPR1), transcript variant 1, mRNA. |
| **OGFRL1** | opioid growth factor receptor-like 1 (OGFRL1), mRNA. |
| **PDAP1** | PDGFA associated protein 1 (PDAP1), mRNA. |
| **PDE4DIP** | phosphodiesterase 4D interacting protein (myomegalin) (PDE4DIP), transcript variant 1, mRNA. |
| **PELI1** | pellino homolog 1 (Drosophila) (PELI1), mRNA. |
| **PEX11B** | peroxisomal biogenesis factor 11 beta (PEX11B), mRNA. |
| **PGPEP1** | pyroglutamyl-peptidase I (PGPEP1), mRNA. |
| **PGRMC1** | progesterone receptor membrane component 1 (PGRMC1), mRNA. |
| **PIK4CB** |  |
| **PLEKHC1** |  |
| **PLXNB2** | plexin B2 (PLXNB2), mRNA. |
| **POLR2J** | polymerase (RNA) II (DNA directed) polypeptide J, 13.3kDa (POLR2J), mRNA. |
| **PON2** | paraoxonase 2 (PON2), transcript variant 1, mRNA. |
| **POU6F1** | POU class 6 homeobox 1 (POU6F1), mRNA. |
| **PPARD** | peroxisome proliferator-activated receptor delta (PPARD), transcript variant 1, mRNA. |
| **PPIB** | peptidylprolyl isomerase B (cyclophilin B) (PPIB), mRNA. |
| **PRKAR2B** | protein kinase, cAMP-dependent, regulatory, type II, beta (PRKAR2B), mRNA. |
| **PRKWNK1** | |
| **PRLR** | prolactin receptor (PRLR), mRNA. |
| **PRSS25** |  |
| **PSME3** | proteasome (prosome, macropain) activator subunit 3 (PA28 gamma; Ki) (PSME3), transcript variant 1, mRNA. |
| **PVR** | poliovirus receptor (PVR), transcript variant 4, mRNA. |
| **RAB1** |  |
| **RAB12** | RAB12, member RAS oncogene family (RAB12), mRNA. |
| **RAB14** | RAB14, member RAS oncogene family (RAB14), mRNA. |
| **RAB7** |  |
| **RAD23B** | RAD23 homolog B (S. cerevisiae) (RAD23B), mRNA. |
| **RAP1B** | RAP1B, member of RAS oncogene family (RAP1B), transcript variant 1, mRNA. |
| **RBBP9** | retinoblastoma binding protein 9 (RBBP9), mRNA. |
| **RBP1** | retinol binding protein 1, cellular (RBP1), mRNA. |
| **RNF14** | ring finger protein 14 (RNF14), transcript variant 5, mRNA. |
| **RNU3IP2** |  |
| **SCP2** | sterol carrier protein 2 (SCP2), transcript variant 2, mRNA. |
| **SDAD1** | SDA1 domain containing 1 (SDAD1), mRNA. |
| **SDC4** | syndecan 4 (SDC4), mRNA. |
| **SDCBP** | syndecan binding protein (syntenin) (SDCBP), transcript variant 2, mRNA. |
| **SDF4** | stromal cell derived factor 4 (SDF4), mRNA. |
| **SDHC** | succinate dehydrogenase complex, subunit C, integral membrane protein, 15kDa (SDHC), nuclear gene encoding mitochondrial protein, transcript variant 4, mRNA. |
| **SEC13L1** |  |
| **SEPP1** | selenoprotein P, plasma, 1 (SEPP1), transcript variant 1, mRNA. |
| **SERINC3** | serine incorporator 3 (SERINC3), transcript variant 2, mRNA. |
| **SFRS3** | splicing factor, arginine/serine-rich 3 (SFRS3), mRNA. |
| **SGTA** | small glutamine-rich tetratricopeptide repeat (TPR)-containing, alpha (SGTA), mRNA. |
| **SLC25A12** | solute carrier family 25 (mitochondrial carrier, Aralar), member 12 (SLC25A12), nuclear gene encoding mitochondrial protein, mRNA. |
| **SLC4A2** | solute carrier family 4, anion exchanger, member 2 (erythrocyte membrane protein band 3-like 1) (SLC4A2), mRNA. |
| **SLC6A8** | solute carrier family 6 (neurotransmitter transporter, creatine), member 8 (SLC6A8), mRNA. |
| **SNRP1C** |  |
| **SNX12** | sorting nexin 12 (SNX12), mRNA. |
| **SNX16** | sorting nexin 16 (SNX16), transcript variant 1, mRNA. |
| **SOX4** | SRY (sex determining region Y)-box 4 (SOX4), mRNA. |
| **SPIRE1** | spire homolog 1 (Drosophila) (SPIRE1), transcript variant 2, mRNA. |
| **SPNA2** |  |
| **SPRED1** | sprouty-related, EVH1 domain containing 1 (SPRED1), mRNA. |
| **STAG2** | stromal antigen 2 (STAG2), transcript variant 1, mRNA. |
| **STARD10** | StAR-related lipid transfer (START) domain containing 10 (STARD10), mRNA. |
| **STAT6** | signal transducer and activator of transcription 6, interleukin-4 induced (STAT6), mRNA. |
| **STRN3** | striatin, calmodulin binding protein 3 (STRN3), transcript variant 2, mRNA. |
| **STX4A** |  |
| **SUI1-RS1** |  |
| **TEGT** |  |
| **TEX10** | testis expressed 10 (TEX10), mRNA. |
| **TGFB1I4** |  |
| **THRA** | thyroid hormone receptor, alpha (erythroblastic leukemia viral (v-erb-a) oncogene homolog, avian) (THRA), transcript variant 2, mRNA. |
| **TIMM17A** | translocase of inner mitochondrial membrane 17 homolog A (yeast) (TIMM17A), nuclear gene encoding mitochondrial protein, mRNA. |
| **TMEM1** | transmembrane protein 1 (TMEM1), transcript variant 1, mRNA. |
| **TMEM50A** | transmembrane protein 50A (TMEM50A), mRNA. |
| **TMEM77** | transmembrane protein 77 (TMEM77), mRNA. |
| **TOMM70A** | translocase of outer mitochondrial membrane 70 homolog A (S. cerevisiae) (TOMM70A), nuclear gene encoding mitochondrial protein, mRNA. |
| **TPRKB** | TP53RK binding protein (TPRKB), mRNA. |
| **TRIM27** | tripartite motif-containing 27 (TRIM27), mRNA. |
| **TRIM41** | tripartite motif-containing 41 (TRIM41), transcript variant 2, mRNA. |
| **TTC13** | tetratricopeptide repeat domain 13 (TTC13), mRNA. |
| **TUBA1** |  |
| **TUBA4** | tubulin, alpha 4 (TUBA4), mRNA. |
| **TUSC4** | tumor suppressor candidate 4 (TUSC4), mRNA. |
| **TXN1** |  |
| **UBE1DC1** | ubiquitin-activating enzyme E1-domain containing 1 (UBE1DC1), transcript variant 1, mRNA. |
| **UBE2Q** |  |
| **UBL3** | ubiquitin-like 3 (UBL3), mRNA. |
| **UFD1L** | ubiquitin fusion degradation 1 like (yeast) (UFD1L), transcript variant 2, mRNA. |
| **UMPS** | uridine monophosphate synthetase (orotate phosphoribosyl transferase and orotidine-5'-decarboxylase) (UMPS), mRNA. |
| **USP20** | ubiquitin specific peptidase 20 (USP20), transcript variant 2, mRNA. |
| **VARS2L** |  |
| **VDAC2** | voltage-dependent anion channel 2 (VDAC2), mRNA. |
| **YWHAG** | tyrosine 3-monooxygenase/tryptophan 5-monooxygenase activation protein, gamma polypeptide (YWHAG), mRNA. |
| **ZDHHC3** | zinc finger, DHHC-type containing 3 (ZDHHC3), mRNA. |
| **ZFP148** |  |
| **ZFP513** |  |
