# Supplementary material for: Transcriptome of Extracellular Vesicles Released by Hepatocytes
Source: PLoS One. 2013 Jul 11;8(7):e68693. doi: 10.1371/journal.pone.0068693 (PMC3708910; doi:10.1371/journal.pone.0068693)
Supplement: Table S5 — Transcripts common to different published list of EVs mRNA, MLP29 and RH. (DOC) [file pone.0068693.s009.doc]

**Table S5. Transcripts common to different published list of EVs mRNA, MLP29 and RH.**

| **SYMBOL** | **DEFINITION** |
| --- | --- |
| **ACTB** | actin, beta (ACTB), mRNA. |
| **COX5B** | cytochrome c oxidase subunit Vb (COX5B), mRNA. |
| **EEF1A1** | eukaryotic translation elongation factor 1 alpha 1 (EEF1A1), mRNA. |
| **EEF2** | eukaryotic translation elongation factor 2 (EEF2), mRNA. |
| **EIF5A** | eukaryotic translation initiation factor 5A (EIF5A), mRNA. |
| **HMGN1** | high-mobility group nucleosome binding domain 1 (HMGN1), mRNA. |
| **MORF4L1** | mortality factor 4 like 1 (MORF4L1), transcript variant 2, mRNA. |
| **NACA** | nascent-polypeptide-associated complex alpha polypeptide (NACA), mRNA. |
| **NME2** | non-metastatic cells 2, protein (NM23B) expressed in (NME2), transcript variant 2, mRNA. |
| **PTP4A2** | PREDICTED: protein tyrosine phosphatase type IVA, member 2, transcript variant 4 (PTP4A2), mRNA. |
| **RPL19** | ribosomal protein L19 (RPL19), mRNA. |
| **RPL21** | ribosomal protein L21 (RPL21), mRNA. |
| **RPL22** | ribosomal protein L22 (RPL22), mRNA. |
| **RPL23** | ribosomal protein L23 (RPL23), mRNA. |
| **RPL24** | ribosomal protein L24 (RPL24), mRNA. |
| **RPL27** | ribosomal protein L27 (RPL27), mRNA. |
| **RPL3** | ribosomal protein L3 (RPL3), transcript variant 2, mRNA. |
| **RPL30** | ribosomal protein L30 (RPL30), mRNA. |
| **RPL31** | ribosomal protein L31 (RPL31), transcript variant 1, mRNA. |
| **RPL35** | ribosomal protein L35 (RPL35), mRNA. |
| **RPL39** | ribosomal protein L39 (RPL39), mRNA. |
| **RPL41** | ribosomal protein L41 (RPL41), transcript variant 2, mRNA. |
| **RPL9** | ribosomal protein L9 (RPL9), transcript variant 2, mRNA. |
| **RPLP1** | ribosomal protein, large, P1 (RPLP1), transcript variant 1, mRNA. |
| **RPS10** | ribosomal protein S10 (RPS10), mRNA. |
| **RPS11** | ribosomal protein S11 (RPS11), mRNA. |
| **RPS12** | ribosomal protein S12 (RPS12), mRNA. |
| **RPS14** | ribosomal protein S14 (RPS14), transcript variant 2, mRNA. |
| **RPS15** | ribosomal protein S15 (RPS15), mRNA. |
| **RPS16** | ribosomal protein S16 (RPS16), mRNA. |
| **RPS2** | ribosomal protein S2 (RPS2), mRNA. |
| **RPS21** | ribosomal protein S21 (RPS21), mRNA. |
| **RPS24** | ribosomal protein S24 (RPS24), transcript variant 2, mRNA. |
| **RPS25** | ribosomal protein S25 (RPS25), mRNA. |
| **RPS26** | ribosomal protein S26 (RPS26), mRNA. |
| **RPS27A** | ribosomal protein S27a (RPS27A), mRNA. |
| **RPS4X** | ribosomal protein S4, X-linked (RPS4X), mRNA. |
| **RPS6** | ribosomal protein S6 (RPS6), mRNA. |
| **RPS7** | ribosomal protein S7 (RPS7), mRNA. |
| **SLC25A3** | solute carrier family 25 (mitochondrial carrier; phosphate carrier), member 3 (SLC25A3), nuclear gene encoding mitochondrial protein, transcript variant 4, mRNA. |
| **SOD1** | superoxide dismutase 1, soluble (SOD1), mRNA. |
| **UBB** | ubiquitin B (UBB), mRNA. |
